# Assessing ChatGPT’s Potential in HIV Prevention Communication: A Comprehensive Evaluation of Accuracy, Completeness, and Inclusivity

**DOI:** 10.1007/s10461-024-04391-2

**Published:** 2024-06-05

**Authors:** Andrea De Vito, Agnese Colpani, Giulia Moi, Sergio Babudieri, Andrea Calcagno, Valeria Calvino, Manuela Ceccarelli, Gianmaria Colpani, Gabriella d’Ettorre, Antonio Di Biagio, Massimo Farinella, Marco Falaguasta, Emanuele Focà, Giusi Giupponi, Adriano José Habed, Wigbertson Julian Isenia, Sergio Lo Caputo, Giulia Marchetti, Luca Modesti, Cristina Mussini, Giuseppe Nunnari, Stefano Rusconi, Daria Russo, Annalisa Saracino, Pier Andrea Serra, Giordano Madeddu

**Affiliations:** 1 Unit of Infectious Diseases, Department of Medicine, Surgery, and Pharmacy, University of Sassari, Sassari, 07100 Italy; 2 Unit of Infectious Diseases, Department of Medical Sciences, University of Turin, Torino, Italy; 3 Associazione Nazionale per la Lotta contro l’AIDS (ANLAIDS), Rome, Italy; 4 Unit of Infectious Diseases, School of Medicine and Surgery, “Kore” University of Enna, Enna, Italy; 5 Department of Media and Culture Studies, Utrecht University, Utrecht, Netherlands; 6 Unit of Infectious Diseases, Department of Public Health and Infectious Diseases, Azienda Policlinico Umberto I, Rome, Italy; 7 Infectious Diseases, San Martino Hospital Genoa, University of Genoa, Genoa, Italy; 8 Mario Mieli, LGBTQIA+ cultural association, Rome, Italy; 9 Associazione Nazionale per la Lotta contro l’AIDS (ANLAIDS), Padova, Italy; 10 Unit of Infectious and Tropical Diseases, Department of Clinical and Experimental Sciences, University of Brescia and ASST Spedali Civili di Brescia, Brescia, Italy; 11 Lega italiana per la lotta contro l’AIDS (LILA), Brescia, Italy; 12 Department of Anthropology, University of Amsterdam, Amsterdam, Netherlands; 13 S.C. Malattie Infettive, Dipartimento di Scienze Mediche e Chirurgiche, University of Foggia, Foggia, Italy; 14 Clinic of Infectious Diseases, Department of Health Sciences, ASST Santi Paolo e Carlo, University of Milan, Milan, Italy; 15 Conigli Bianchi, Artivists against Serophobia, Italy; 16 University of Modena and Reggio Emilia, Modena, Italy; 17 Unit of Infectious Diseases, Department of Clinical and Experimental Medicine, ARNAS Garibaldi Hospital, University of Catania, Catania, Italy; 18 Infectious Diseases Unit, Ospedale Civile di Legnano, ASST Ovest Milanese, DIBIC Luigi Sacco, Università degli Studi di Milano, Legnano, 20025 Italy; 19 Network Persone Sieropositive (NPS), Rome, Italy; 20 Clinic of Infectious Diseases, Department of Precision and Regenerative Medicine and Ionian Area—(DiMePRe-J), University of Bari “Aldo Moro”, Bari, Italy; 21 Department of Medicine, Surgery and Pharmacy, University of Sassari, Sassari, Italy; 22 PhD School in Biomedical Science, Biomedical Science Department, University of Sassari, Sassari, Italy

**Keywords:** HIV, PrEP, AI, LLM, ChatGPT, HIV transmission

## Abstract

With the advancement of artificial intelligence(AI), platforms like ChatGPT have gained traction in different fields, including Medicine. This study aims to evaluate the potential of ChatGPT in addressing questions related to HIV prevention and to assess its accuracy, completeness, and inclusivity. A team consisting of 15 physicians, six members from HIV communities, and three experts in gender and queer studies designed an assessment of ChatGPT. Queries were categorized into five thematic groups: general HIV information, behaviors increasing HIV acquisition risk, HIV and pregnancy, HIV testing, and the prophylaxis use. A team of medical doctors was in charge of developing questions to be submitted to ChatGPT. The other members critically assessed the generated responses regarding level of expertise, accuracy, completeness, and inclusivity. The median accuracy score was 5.5 out of 6, with 88.4% of responses achieving a score ≥ 5. Completeness had a median of 3 out of 3, while the median for inclusivity was 2 out of 3. Some thematic groups, like behaviors associated with HIV transmission and prophylaxis, exhibited higher accuracy, indicating variable performance across different topics. Issues of inclusivity were identified, notably the use of outdated terms and a lack of representation for some communities. ChatGPT demonstrates significant potential in providing accurate information on HIV-related topics. However, while responses were often scientifically accurate, they sometimes lacked the socio-political context and inclusivity essential for effective health communication. This underlines the importance of aligning AI-driven platforms with contemporary health communication strategies and ensuring the balance of accuracy and inclusivity.

**Supplementary Information:**

The online version contains supplementary material available at 10.1007/s10461-024-04391-2.

## Introduction

ChatGPT is an artificial intelligence (AI) large language model (LLM) implemented by OpenAI as an advanced conversational AI technology [1]. It is based on the GPT (Generative Pre-trained Transformer) architecture, specifically GPT 4.0, and is designed to generate human-like responses to text-based input. The model has been trained on a massive amount of diverse text data from the Internet, enabling it to understand language patterns and context [2, 3].

ChatGPT employs deep learning techniques, specifically transformer neural networks, which enable it to understand and generate coherent, contextually relevant responses in natural language [4]. It has the ability to comprehend questions, statements, and prompts. In addition, it can generate appropriate answers, suggestions, and explanations or engage in conversation on a wide range of topics [5]. One notable feature of ChatGPT is its ability to provide personalized responses [2, 6, 7].

The GPT is a deep learning architecture widely used for various natural language processing (NLP) tasks. It was first introduced by OpenAI in 2018 and has since undergone several iterations, with GPT.0 being the most recent and advanced version [8]. The GPT architecture is based on the Transformer model, a neural network that utilizes self-attention mechanisms to process sequential data, such as sentences or paragraphs. The Transformer model revolutionized NLP by addressing the limitations of recurrent neural networks (RNNs) in capturing long-range dependencies and enabling parallel processing [2]. In the case of GPT, the “pre-trained” aspect refers to the model being initially trained on a large corpus of text data [3]. The pre-training phase involves unsupervised learning, where the model learns to predict the next word in a sentence or fill in missing words based on the surrounding context. By training on a massive amount of text, GPT is exposed to a wide range of language patterns and gains an understanding of syntax, semantics, and general world knowledge. Once pre-training is complete, the model is fine-tuned on specific downstream tasks, such as text classification, question answering, or language translation. During fine-tuning, the model is trained on labelled data specific to the task at hand, allowing it to adapt its pre-learned knowledge to the particular task’s requirements. GPT models, including GPT-4.0, have shown high performance on language tasks, demonstrating their ability to generate coherent and contextually appropriate text. They excel in text completion, text generation, and language understanding [6].

Recently, ChatGPT has received attention from researchers from different medical disciplines [9–11]. For example, Dave et al. outline the use of ChatGPT in producing scientific literature to assist in researching and writing scientific papers. This capability makes it easier for researchers to draft manuscripts and significantly reduces the time spent searching for and selecting relevant articles, allowing more time for actual research work and methodology [9]. In addition, questions are beginning to be asked about the revolution artificial intelligence can introduce into diagnostics and clinical practice [10–13]. For example, Chiesa-Estomba et al. found that ChatGPT is a promising tool in the clinical decision-making process within the salivary gland clinic, particularly for candidates for sialendoscopy treatment [14]. On the contrary, Dave et al. point out the limitations of ChatGPT use in the medical field. They highlight ethical and legal concerns, including potential legal issues such as copyright infringement and accuracy or bias in generated content. These limitations underscore the importance of human oversight and the need to address issues such as the quality and nature of the training data, which can significantly influence the accuracy of AI-generated text [9].

In the context of infectious diseases, Cheng et al. explored the role of ChatGPT in providing precise, up-to-date information to the general public, healthcare professionals, and policymakers [15]. One potential implementation could be giving information about HIV prevention. However, the risk of disseminating false, inaccurate, or incomplete information could increase risky behavior, causing significant harm to individuals’ health [16].

With this analysis, we aim to evaluate the possible uses and constraints of ChatGPT, providing perspective on the employment of AI for communicating information related to HIV and its prevention. This research thus contributes to current debates on incorporating AI-driven tools in healthcare practices.

## Methods

We aimed to investigate the potential applications of AI, specifically ChatGPT, in giving information about HIV and its prevention to the general population and to assess how accurate, complete, and inclusive the answers produced are. To address these questions, we developed a structured framework that combines interdisciplinary collaboration with systematic data collection and analysis.

### Teams

We created a collaborative team that involved 15 doctors, six members of HIV communities, and three experts in gender and queer studies. The members of HIV communities are individuals who are either living with HIV, engaged in advocacy or support roles within HIV-focused organizations, or actively participate in community-based initiatives aimed at HIV awareness, prevention, and care. These members bring their insights and experience, offering perspectives that differ from those of the medical professionals involved in our research. They contribute unique insights from lived experiences, essential for a holistic understanding of HIV prevention and care. Their perspectives combine with the clinical and scientific viewpoints doctors provide, enriching the study with a broader diversity of thought and experience.

For the purposes of this study, the participating researchers were divided into five working teams. The first team included three resident doctors working in the HIV field. Three additional teams comprised four Infectious Diseases professors with long-standing experience in HIV and two HIV community members. A fifth team was composed of three gender and queer studies experts.

This division was designed to ensure comprehensive coverage of the diverse areas of HIV prevention, including general information about HIV, behaviors increasing the risk of contracting HIV, HIV and pregnancy, HIV testing, and the use of prophylaxis.

## Measures

### Questions Development and Answers Collection

The team one was in charge of formulating questions about the topics mentioned above. To do this, the team examined HIV fora and authoritative websites [17–19]. In addition, the team created other questions based on their experience in counselling people on these topics [8]. The questions were then submitted to ChatGPT 4, and the answers were collected in Excel. Questions were grouped into five topics: (1) general information about HIV (20 questions); (2) behaviors increasing the risk of contracting HIV (54 questions); (3) pregnancy and HIV (8 questions); (4) testing (12 questions); (5) use of prophylaxis (36 questions). The questions were formulated, and the answers were collected between 10 June 2023 and 22 June 2023.

### Questions and Answers Evaluation

All responses from ChatGPT were meticulously recorded and provided to teams 2, 3, and 4. A designated team member (team one) oversaw the recording and transcription of all dialogues between ChatGPT and the staff members inputting questions, ensuring the fidelity of the information captured in a comprehensive file, which was then sent to the other teams. Team two reviewed the answers related to “general information about HIV” (topic 1, 20 questions) and “testing” (topic 4, 12 questions). Team three addressed “behaviors increasing the risk of contracting HIV” (topic 2, 54 questions). Team four evaluated “pregnancy and HIV” (topic 3, 8 questions) and “use of prophylaxis” (topic 5, 36 questions). Their task was to read the questions created by the first team and rate them in terms of level of expertise (LOE) (low, medium, high). A “low LOE” question was defined as one that every medical doctor should be able to answer. A “medium LOE” question was one that an infectious diseases specialist should be qualified to handle. A “high LOE” question was intended for those with specific expertise in HIV. If the median score from the six evaluators was 1.5 or 2.5, an additional researcher, external to the team, assessed the question to determine its LOE level.

Additionally, the three teams reviewed the answers generated by ChatGPT. They judged them based on two aspects: (i) accuracy: a six-point Likert scale was employed, with 1 representing a completely incorrect response, 2 denoting the presence of more incorrect than correct elements, 3 indicating an equal balance of correct and incorrect elements, 4 denoting the presence of more correct than incorrect elements, 5 for an almost fully correct response, and 6 for an entirely correct response; (ii) completeness: a three-point Likert scale was used, where 1 stood for an incomplete answer that only addressed some aspects of the question with significant parts missing or incomplete, 2 represented an adequate answer that addressed all aspects of the question and provided the minimum information required for completeness, and 3 denoted a comprehensive response that covered all aspects of the question and offered additional information or context beyond expectations.

Furthermore, these four teams were supported by three gender and queer studies experts (team five) who assessed the language used by ChatGPT, rating it based on inclusivity. For this evaluation, we used a three-point Likert scale: 1 representing an openly exclusionary/offensive/stigmatizing answer, 2 denoting an answer that used incorrect terms that are potentially exclusionary and/or stigmatizing, and 3 for an answer that used a fully inclusive and non-stigmatizing language. We referred to the National Institute of Allergy and infectious diseases guide for the terms used [20]. In addition, we did not follow the principle of cumulative effect (if an answer had several minor problems, which would qualify it as 2 on the Likert scale, we rated it 2 as we did for answers with only one issue).

Researchers were asked not to express judgment if they did not know the topic and could not evaluate the answer.

The expert teams have performed the evaluation between 23 June 2023 and 20 August 2023.

The complete evaluation is available in Supplementary Materials S2.

### Statistical Analysis

Categorical variables were reported in numerals and percentages of the total. Descriptive statistics for quantitative variables were given as the median (interquartile range (IQR)). Differences in accuracy and completeness scores between groups of different LOE and distinct question categories were assessed using the Kruskal-Wallis test. The differences in the proportion of correct dichotomous responses among various subgroups were assessed using the Chi-square or Fisher exact test. The level of statistical significance was set at *p* < 0.05. Statistical analysis was performed using STATA 16.1 (StataCorp, Texas, United States).

#### Ethical considerations

The ethical review and approval requirement was waived because the study did not include any analysis of humans or animals.

## Results

Overall, 130 different questions were designed to be submitted to ChatGPT. We used ChatGPT version 4 on 11 June 2023 to perform this study [8]. The complete set of clinical questions is presented In Supplementary Table 1. The questions and answers were evaluated for questions’ LOE, accuracy, and completeness by three teams of experts in HIV and HIV community members. A team of gender and queer studies experts assessed the degree of inclusivity of the answers.

Based on the 130 questions and answers, we gathered 780 evaluations concerning questions’ LOE, accuracy and completeness, and 130 evaluations of answers’ inclusivity.

In terms of LOE, 38 questions (29.2%) were rated as low, 75 (57.7%) as medium, and 17 (13.1%) as high.

### Accuracy

The overall accuracy median was 5.5 (IQR 5–6) points out of a maximum of six, with the majority of responses reaching a score $\ge$five points (88.4%). Only one answer (“Is it possible to get HIV infection using condoms?”) was evaluated 3. No 1 and 2 points were recorded.

Dividing the questions according to the LOE, no differences were present in the accuracy of the answers (Table 1). Evaluating the five different groups of responses, there was a higher accuracy for the answers about “behaviors increasing the risk of contracting HIV” (topic 2) and “use of prophylaxis” (topic 5) (Table 2).

### Completeness

The median completeness was 3 points (IQR 3–3) out of a possible six. No answer received a score of 1 point, but 31 answers (23.9%) were evaluated with 2 points. There were no statistical differences in completeness across LOE (Table 1) or among the five question groups (Table 2).

**Table 1:** Accuracy, completeness, and inclusivity of 130 answers given by ChatGPT about HIV, divided by the questions’ level of expertise

	Overall (130)	Low level of expertise questions (38)	Medium level of expertise (75)	High level of expertise questions (17)	Chi-square*	*p*-value*
Median (IQR)						
Accuracy	5.5 (5–6)	5.5 (5–6)	5.5 (5–6)	5 (5–6)	1.970	0.373
Completeness	3 (3–3)	3 (3–3)	3 (3–3)	3 (3–3)	0.185	0.912
Inclusivity	2 (2–3)	2.5 (2–3)	2 (2–3)	2 (2–3)	2.008	0.366
N(%)						
Accuracy, 6 points	54 (41.5)	17 (44.7)	27 (36.0)	10 (58.8)	6.265	0.349
Accuracy 5 points	61 (46.9)	16 (42.1)	40 (53.3)	5 (29.4)		
Accuracy 4 points	14 (1.8)	4 (10.5)	8 (10.7)	2 (11.8)		
Accuracy 3 points	1 (0.8)	1 (2.6)	0	0		
Completeness, 3 points	99 (76.1)	28 (73.7)	58 (77.3)	13 (76.5)	0.186	0.956
Completeness, 2 points	31 (23.9)	10 (26.3)	17 (22.7)	4 (23.5)		
Inclusivity 3 points	57 (43.8)	19 (50.0)	33 (44.0)	5 (29.4)	2.024	0.387
Inclusivity 2 points	73 (56.2)	19 (50.0)	42 (56.0)	12 (70.6)		

*We used Kruskal-Wallis equality-of-populations rank test for the continuous variables, and Chi-squared test or Fisher exact test for categorical variables

### Inclusivity

The median inclusivity score was 2 points (IQR 2–3) out of a possible three. The majority of the answers, 73 (56.3%), received a score of 2, while 57 (43.7%) received a score of 3. No answer was deemed overtly exclusionary, offensive, or stigmatizing. There were no statistical differences in scores across LOE.

When examining different areas, only the topic “use of prophylaxis” had a majority of answers, scoring 3 points (61.1%). In contrast, the topics “pregnancy and HIV” and “testing” had only 2 (25.0%) and 1 (8.3%) answers, respectively, evaluated with 3 points (Table 2).

**Table 2:** Accuracy, completeness, and inclusivity of 130 answers given by ChatGPT about HIV, divided by the different question areas

	Overall	Topic 1	Topic 2	Topic 3	Topic 4	Topic 5	Chi-square*	*p*-value*
Median (IQR)								
Accuracy	5.5 (5–6)	5 (5-5.5)	5.5 (5–6)	5.5 (5-5.75)	5.25 (5-5.5)	6 (5.5-6)	19.117	0.001
Completeness	3 (3–3)	3 (2.5-3)	3 (3–3)	3 (2.5-3)	3 (2.5-3)	3 (3–3)	7.247	0.123
Inclusivity	2 (2–3)	2 (2–3)	2 (2–3)	2 (2-2.5)	2 (2–2)	3 (2–3)	11.696	0.020
Number (%)								
Accuracy, 6 points, n(%)	54 (41.5)	1 (5)	24 (44.4)	2 (25)	2 (16.7)	25 (69.4)	33.580	< 0.001
Accuracy 5 points n(%)	61 (46.9)	15 (75)	21 (38.9)	5 (62.5)	10 (83.3)	10 (27.8)		
Accuracy 4 points, n(%)	14 (1.8)	4 (20)	8 (14.8)	1 (12.5)	0	1 (2.8)		
Accuracy 3 points, n(%)	1 (0.8)	0	1 (1.85)	0	0	0		
Completeness, 3 points	99 (76.1)	12 (60.0)	43 (79.6)	6 (75.0)	7 (58.3)	31 (86.1)	7.303	0.107
Completeness, 2 points	31 (23.9)	8 (40.0)	11 (20.4)	2 (25.0)	5 (41.7)	5 (13.9)		
Inclusivity 3 points, n(%)	57 (43.8)	8 (40.0)	24 (44.4)	2 (25.0)	1 (8.3)	22 (61.1)	11.787	0.016
Inclusivity 2 points, n(%)	73 (56.2)	12 (60.0)	30 (55.6)	6 (75.0)	11 (91.7)	14 (38.9)		

Topic 1: General information about HIV; Topic 2: Behaviors increasing the risk of contracting HIV; Topic 3: pregnancy and HIV; Topic 4: Testing; Topic 5: Use of prophylaxis. *We used Kruskal-Wallis equality-of-populations rank test for the continuous variables, and Chi-squared test or Fisher exact test for categorical variables

ChatGPT demonstrated several issues regarding inclusivity, with a few prominent examples highlighted here. A recurring error was the use of terms such as “HIV infection” and “HIV-positive”. The NIAID HIV language guide advises the use of the more inclusive terms “HIV transmission” and “people living with HIV”. Another frequent oversight by ChatGPT was its assumption that people with a vagina are women and those with a penis are men, which overlooks and excludes many transgender and non-binary individuals. In its interactions on anal sex, ChatGPT adopted terms like “top” and “bottom,” predominantly associated with the gay male community, thus suggesting the limited view that only gay men participate in anal sex.

## Discussion

Since its release in November 2022, ChatGPT has quickly become the fastest-growing application, with over 100 million users and over 1.8 billion monthly visits [21]. 

However, assessing the ethical and data privacy concerns related to AI use in healthcare is essential, as well as adequate validation and testing of these systems before their utilization. This may be significant since this information is available to the general public, who may lack the knowledge to assess it critically. This may generate unrealistic expectations, spread misinformation, and/or potentially influence patient-professional relationships. While much research debates the potential advantages and disadvantages of employing ChatGPT in scientific research [22–24], there is a considerable gap in knowledge about its use in clinical settings. Notably, the accuracy of information provided by ChatGPT to health professionals and patients has not been thoroughly investigated.

Moreover, given its free access and user-friendly nature, ChatGPT could become a primary resource for the general public searching for medical information. This poses the potential risk of overshadowing healthcare professionals, especially in areas that continue to be seen as sources of embarrassment for a significant portion of the population, like sexuality and HIV. Previous studies have highlighted a lack of HIV knowledge among the general population [25, 26]; thus, it seems reasonable that people without training may search for information regarding this topic on ChatGPT. Consequently, we decided to assess ChatGPT’s ability to answer questions.

ChatGPT scored well in accuracy and completeness, with most answers being accurate and comprehensive. The median score of 3 (out of 6) suggests that the responses, while accurate, might not always be as comprehensive as one would hope for, especially in the context of medical information where thoroughness can be critical.

When looking deeper into the data, subtle variations become apparent. For instance, while there were no differences in accuracy across questions of varying LOE, there were differences when categorizing questions into different thematic groups. The heightened accuracy in responses about behaviours associated with HIV risk and prophylaxis use suggests that some topics might be better represented or understood than others.

We noted a possible gap with current health education and advocacy methods. The responses, for instance, adhere to a normative script but lack a sex-positive lens. This is not just about promoting accuracy but about embracing a holistic perspective that acknowledges the complexities of human sexuality, choices, and behaviours. Authentic prevention and decisions surrounding safer sex practices are influenced by a myriad of factors that the platform often seems to overlook. The answers tend to prioritize scientific correctness over pedagogical relevance. For example, few answers include a peer education approach, a strategy recognized for its efficacy in modern health communication, making the information delivery appear somewhat outdated [27, 28].

An example of this dichotomy between scientific accuracy and socio-political sensitivity is seen in ChatGPT’s response to a question about HIV transmission through kissing. While the answer was scientifically accurate in stating that the risk is extremely low or non-existent and then enumerating hypothetical scenarios for transmission, it missed an essential nuance. The unequivocal message that HIV is not transmitted through kissing has been foundational in combating stigma and misinformation for over four decades [29]. This is not merely a scientific fact but a significant socio-political stance that has been crucial in the history of HIV prevention and advocacy. By not strongly emphasizing this point, the platform might inadvertently perpetuate stigma, even if unintentionally.

These observations underscore the challenges of using AI in healthcare communication. The overall challenge is not merely to be scientifically accurate but also to understand the broader socio-cultural implications of the information being provided and ensure that messages empower, educate, and advocate.

Another point of concern arises when considering that in only 51 out of the 130 responses, ChatGPT recommended consulting a healthcare provider. Such an omission, especially in healthcare, highlights the risk of users depending only on the platform instead of seeking professional advice. However, our findings are inconsistent with the conclusions of other studies investigating the performance of ChatGPT in the HIV field. For example, Yi Koh et al. emphasize that, while being inaccurate in giving answers to questions regarding specific populations (e.g. pregnant women living with HIV), ChatGPT always encourages seeking health care professionals’ assistance [30]. This discrepancy could be due to differences in the sets of questions. While the questions submitted to ChatGPT by Yi Koh et al. were formulated from the perspective of people living with HIV, we focused on prevention; hence, we formulated questions from the perspective of the general population [30].

Concerning inclusivity, ChatGPT effectively uses inclusive language in many of its responses; however, almost all of our questions already utilized neutral, non-stigmatizing terminology. Thus, we cannot evaluate whether ChatGPT mirrors the language employed in the questions it receives or can respond in inclusive and non-stigmatizing manners to differently formulated questions. Moreover, most of our questions were general. They did not address the specific position of minoritized subjects (e.g., none of our questions were formulated from the perspective of a trans woman, a gay man, a black lesbian woman, etc.). ChatGPT responded to these questions with equally generic answers. We do not know whether a more specific or explicitly inclusive question would yield a more precise and inclusive response or would still generate a generic answer. In this regard, the literature is still limited. A recently published article investigated the presence of biases regarding ethnicity and insurance type; the findings suggest no significant discrepancies regarding these domains [31]. However, when tested for gender biases, ChatGPT showed worrying results. Hirani et al. tested ChatGPT by asking ten words associated with men and women respectively: “housewife” and “maid” were reported for women, while “doctor” and “CEO” were reported for men [32]. Even if the question itself was discriminating and excluding non-binary people, and the inclusivity of language was not directly evaluated, this study points out how word embeddings can continue and increase web-based biases.

It is important to note that in its current state, ChatGPT is bounded by its knowledge cutoff, which extends only until September 2021 [4]. However, there is potential on the horizon to overcome this. OpenAI’s recent introduction of application programming interfaces (APIs) and the possibility of developing plugins might soon enable ChatGPT to tap into third-party applications, offering real-time or updated knowledge retrieval [33]. 

Furthermore, ChatGPT’s potential in Medicine needs to be contextualized. While its current expertise is not superior to a trained healthcare professional, it is interesting to consider a future where ChatGPT could access up-to-date data from comprehensive sources such as PubMed or SCOPUS. Such an extensive knowledge base could surpass the informational capacity of any individual professional. However, the critical distinction lies in interpreting and applying this knowledge, a realm where human professionals still hold a clear advantage. Accessing and interpreting AI’s vast “knowledge” could be an invaluable asset for healthcare professionals. However, for the layperson, this poses challenges. The sophistication of an evolved AI might tempt some to sideline traditional medical consultations. We have already observed this phenomenon, with many resorting to Google for healthcare advice [34, 35].

In a recently published study, answers given by ChatGPT and physicians were comparatively evaluated by healthcare professionals. ChatGPT responses were preferred in 78.6% of cases, performing better in the empathy domain (9.8 times more empathy encountered in AI responses when compared to physicians’ answers) [36]. While offering an interesting and unexpected evaluation of ChatGPT’s performance, this study has some flaws; it should be considered that AI lacks the human-to-human interaction advantages that physicians and healthcare providers can benefit from. Counselling with a patient does not just amount to information exchange. Still, it is a dynamic interaction in which healthcare operators should also intercept and interpret patients’ reactions and non-verbal communication to achieve more effective communication and build doctor-patient relationships [36, 37]. While this study highlighted stronger empathy in ChatGPT answers, evaluating the level of empathy achieved during in-person counselling managed by a human being is challenging. As experts in the field, we find that human-to-human interaction is still highly needed when addressing people in general and vulnerable persons in particular, including persons living with HIV [38].

Our study has several limitations. Firstly, the categorization of questions by LOE was subjective, relying on the researchers’ judgment. The criteria used to determine accuracy, completeness, and inclusivity also had an inherent subjective aspect. The teams responsible for evaluating the responses consisted of HIV experts (professors and HIV community members), which raises the question of how understandable ChatGPT’s answers might be for the layperson. Additionally, we posed direct questions, so we are unable to assess how ChatGPT performs in a more extended, back-and-forth conversation. Also, we did not compare the answers the AI gave with answers by physicians and counsellors.

Another notable limitation is ChatGPT’s evolving nature. While we tested the AI after the last update in September 2021, the model’s responses could alter in the future. Lastly, assessing ChatGPT’s reliability is inherently challenging. The lack of established systems for such assessments necessitates devising unique tools. Although our study showed consistent evaluation, this brings subjectivity and potential interrater reliability issues.

## Conclusion

ChatGPT’s ascent as a leading AI platform, notably within the healthcare domain, marks a significant stride in technological innovation with profound implications. Our research indicates that while ChatGPT consistently provides accurate answers to HIV-related questions, it is occasionally inadequate in comprehensiveness and inclusivity, particularly within the delicate sphere of healthcare communication. Its commitment to scientific precision is occasionally overshadowed by its lapses in presenting medically sound and culturally sensitive information. While future iterations of ChatGPT may incorporate real-time data updating capabilities, they are not a replacement for real medical consultations. Increased educational initiatives are essential to guide the public on responsibly utilizing the capabilities of a potent tool like ChatGPT.

### Electronic Supplementary Material

Below is the link to the electronic supplementary material.

Supplementary Material 1

Supplementary Material 2

## Data Availability

The data that supports the findings of this study are available in the supplementary material of this article.
