# Supplementary material for: Assessing ChatGPT’s Potential in HIV Prevention Communication: A Comprehensive Evaluation of Accuracy, Completeness, and Inclusivity
Source: AIDS Behav. 2024 Jun 5;28(8):2746–54. doi: 10.1007/s10461-024-04391-2 (PMC11286632; doi:10.1007/s10461-024-04391-2)
Supplement: Supplementary file 1 — Supplementary Material 1 [file 10461_2024_4391_MOESM1_ESM.docx]

Supplemental Material 1. List of the 130 Questions and Answers.

| 1. What is HIV? | |
| --- | --- |
| HIV stands for Human Immunodeficiency Virus. It's a virus that attacks the body's immune system, specifically the CD4 cells (T cells), which help the immune system fight off infections. If left untreated, HIV can reduce the number of these cells in the body, making it harder to fight off infections and disease.  Over time, HIV can lead to AIDS (Acquired Immune Deficiency Syndrome), which is the most severe stage of HIV infection. People with AIDS have such badly damaged immune systems that they get an increasing number of severe illnesses, which are known as opportunistic infections.  HIV is primarily transmitted through certain body fluids—blood, semen, pre-seminal fluids, rectal fluids, vaginal fluids, and breast milk. These fluids must come into contact with a mucous membrane or damaged tissue or be directly injected into the bloodstream (from a needle or syringe) for transmission to occur.  As of my knowledge cutoff in September 2021, there is no cure for HIV, but it can be controlled with proper medical care, including antiretroviral therapy (ART). If taken as prescribed, these medications can keep people with HIV healthy for many years and greatly reduce the chance of transmitting the virus to sex partners.  HIV testing is important to know your status and to start treatment early if needed. Preventive measures include using condoms during sex, never sharing needles, and using pre-exposure prophylaxis (PrEP) if you're at high risk of HIV. Regular testing for HIV and other STIs is also important if you're sexually active, especially if you have multiple sexual partners or are in a relationship with someone who has HIV.  For the most up-to-date information on HIV/AIDS, consult trusted health organizations or medical professionals. |  |
| 1. What is AIDS? | |
| AIDS stands for Acquired Immunodeficiency Syndrome. It is the most severe stage of HIV (Human Immunodeficiency Virus) infection.  HIV is a virus that attacks the body's immune system, specifically the CD4 cells, which are a type of white blood cell that plays a major role in protecting the body from disease. Over time, as more and more CD4 cells are destroyed by the virus and the count falls below a certain level, the body becomes less able to fight off infections and diseases.  When the immune system becomes severely damaged and the individual becomes susceptible to certain types of infections and illnesses known as opportunistic infections, this stage of the infection is classified as AIDS.  Opportunistic infections take advantage of a weakened immune system and can cause severe illnesses. These illnesses can be a wide range of conditions, including pneumocystis pneumonia, tuberculosis, severe bacterial infections, and certain types of cancer, among others.  As of my knowledge cutoff in September 2021, there is no cure for HIV/AIDS, but it can be managed effectively with antiretroviral therapy (ART). If taken as prescribed, these medications can keep people with HIV healthy for many years and greatly reduce their chance of transmitting the virus to others.  Early diagnosis and treatment of HIV is key to preventing progression to AIDS. Regular HIV testing and safe practices, such as using condoms during sex and not sharing needles, are vital to prevent HIV transmission.  It's also important to note that people living with HIV/AIDS should be treated with respect and understanding, and stigma and discrimination against them should be combated at all levels of society. For the most up-to-date information on HIV/AIDS, consult trusted health organizations or medical professionals. |  |
| 1. What are the symptoms of HIV? | |
| HIV infection progresses in stages, and the symptoms can vary widely depending on the stage and individual.   1. Acute HIV infection: Some people may experience a flu-like illness within 2 to 4 weeks after infection (although some people may feel healthy and may not notice any symptoms). Symptoms can last from a few days to several weeks and may include:    - Fever    - Chills    - Rash    - Night sweats    - Muscle aches    - Sore throat    - Fatigue    - Swollen lymph nodes    - Mouth ulcers   These symptoms are the body's natural response to the virus and are similar to those seen in other viral infections. Not everyone will get these symptoms, and some people may not have any symptoms at all for 10 years or more.   1. Clinical latency (HIV inactivity or dormancy): This period can last a decade or longer, during which the virus is still active but reproduces at much lower rates. Some people may have persistent swollen lymph nodes during this period. 2. Progression to AIDS: If HIV is not treated, it will eventually progress to AIDS, the final stage of infection. Symptoms can include:    - Rapid weight loss    - Recurring fever or profuse night sweats    - Extreme and unexplained tiredness    - Prolonged swelling of the lymph glands in the armpits, groin, or neck    - Sores of the mouth, anus, or genitals    - Pneumonia    - Memory loss, depression, or other neurological disorders   Each of these symptoms can also be related to other illnesses. The only sure way to determine whether you are infected with HIV is to be tested for HIV.  Antiretroviral therapy (ART) can control the virus and help prevent transmission so that people with HIV, and those at substantial risk, can enjoy healthy, long and high-quality lives. If you are concerned about possible infection, it's important to get tested and to speak to a healthcare provider. |  |
| 1. May I identify people with HIV by their physical aspects? | |
| No, you cannot reliably identify someone with HIV based on their physical appearance. The symptoms of HIV vary widely between individuals and many people with HIV may not show any physical symptoms, especially in the early stages of the infection.  People with HIV can look healthy and feel well. It's also worth noting that many symptoms associated with HIV, such as weight loss, fever, or night sweats, could be due to a number of other conditions, not just HIV.  Moreover, antiretroviral therapy, the standard treatment for HIV, helps people with the virus to live healthy lives without developing the symptoms or illnesses associated with the final stage of the infection, AIDS (Acquired Immune Deficiency Syndrome).  The only sure way to know if someone has HIV is through an HIV test. It's important to remember that HIV is not transmitted through casual contact such as shaking hands, hugging, or sharing dishes, so there is no need to know someone's HIV status unless you plan to engage in behaviors that could transmit the virus, such as sexual activity or sharing needles.  Lastly, it's worth emphasizing that stigmatizing or discriminating against people with HIV is harmful and unproductive. HIV is a medical condition and should be treated as such. Everyone deserves respect and understanding, regardless of their HIV status. |  |
| 1. How a person with HIV looks like? | |
| People with HIV can look like anyone else. They can be of any age, gender, race, or body type. There are no specific physical characteristics or visible symptoms that can reliably indicate whether a person has HIV.  In the early stages of the disease, people with HIV often show no symptoms and appear healthy. Even in the later stages, symptoms can vary widely and can be similar to those of other illnesses.  In some cases, people with advanced HIV disease, also known as AIDS, may lose weight or appear sickly due to opportunistic infections or cancers that the body can no longer effectively fight. However, with effective antiretroviral therapy (ART), many people with HIV are able to manage the virus and live healthy, active lives.  The only sure way to know if someone has HIV is through testing. It's important to get tested if you're at risk, and to use preventive measures like condoms and pre-exposure prophylaxis (PrEP) to protect against HIV.  Stigma and discrimination related to HIV and AIDS have caused a lot of harm over the years. It's essential to treat everyone with respect and dignity, regardless of their HIV status, and to combat stigma and misinformation about HIV and AIDS. |  |
| 1. How can I tell if I have HIV? | |
| It's important to know that you cannot rely on symptoms alone to know whether you have HIV. Many people who are infected with HIV don't have any symptoms at all for several years. Others may experience a flu-like illness within 2-4 weeks after exposure (known as acute HIV infection), which may include fever, headache, tiredness, swollen lymph nodes, joint pain, muscle aches, sore throat, rash, night sweats, and/or mouth or genital ulcers. These symptoms can last anywhere from a few days to several weeks.  However, these symptoms are not unique to HIV and could be caused by other illnesses or conditions. Additionally, even if you feel fine, you could still be infected with HIV and be able to transmit the virus to others.  The only reliable way to know whether you have HIV is to get tested. HIV tests are typically performed on blood or oral fluid, and they look for antibodies or antigens (proteins produced by the immune system in response to the virus) or for the virus itself.  If you believe you may have been exposed to HIV, or if you're sexually active and have never been tested for HIV, it's a good idea to speak with a healthcare provider about getting tested. Regular HIV testing is important for everyone, but it's particularly important for people who have multiple sexual partners, men who have sex with men, people who use intravenous drugs, and people with other risk factors for HIV.  If you test positive for HIV, it's important to start treatment as soon as possible. With proper treatment, people with HIV can live long, healthy lives and significantly reduce their risk of transmitting the virus to others. |  |
| 1. How can HIV be transmitted? | |
| HIV is transmitted from person to person through certain body fluids. These include:   1. Blood 2. Semen (cum) and pre-seminal fluid (pre-cum) 3. Rectal fluids 4. Vaginal fluids 5. Breast milk   HIV is not transmitted through saliva, tears, sweat, feces, or urine.  Here are the main ways that HIV can be transmitted:   1. **Unprotected Sex**: Having anal or vaginal sex without using a condom or taking medicines to prevent or treat HIV with someone who has the virus is the most common way HIV is transmitted. The virus can be present in semen, vaginal fluid, and rectal fluid. 2. **Sharing Needles or Syringes**: Sharing needles or syringes, rinse water, or other equipment (works) used to prepare drugs for injection with someone who has HIV. The virus can be present in the blood on used needles. 3. **From mother to child**: A mother can pass HIV to her baby during pregnancy, childbirth, or breastfeeding. 4. **Blood Transfusions and Organ/Tissue Transplants**: It is possible, but very rare, to get HIV from receiving a blood transfusion or organ/tissue transplant that is infected with the virus. In many countries, blood donations are routinely screened for HIV.   HIV cannot be transmitted through casual contact, such as shaking hands, hugging, or sharing dishes, or through insect bites like mosquitoes.  Practices like using condoms correctly and consistently, not sharing needles or syringes, and taking pre-exposure prophylaxis (PrEP) if you are at high risk, can significantly reduce the risk of HIV transmission. Regular testing is also key to knowing your status and preventing the spread of HIV. If you are living with HIV, taking antiretroviral therapy (ART) as prescribed can reduce the amount of virus in your body (viral load) to undetectable levels, which means you can't transmit the virus to others through sex, also known as "U=U" or "Undetectable equals Untransmittable". |  |
| 1. What are the sexual practices at risk of HIV? | |
| Engaging in certain sexual behaviors can increase your risk of acquiring or transmitting HIV. These include:   1. **Unprotected Sex:** This is the most common way HIV is transmitted. Unprotected sex means having anal, vaginal, or oral sex without using a condom. The risk is greater for anal sex because the lining of the rectum is thin and may allow the virus to enter the body during sex. 2. **Multiple Sexual Partners:** Having many partners increases the risk of HIV because it raises the chance of coming into contact with someone who is infected. It's also more difficult to ensure that condoms are used consistently and correctly with multiple partners. 3. **Having Other Sexually Transmitted Infections (STIs):** Certain STIs, like syphilis, herpes, and gonorrhea, can make you more susceptible to HIV infection. These diseases can cause sores or breaks in the skin or lining of the mouth, vagina, penis, or rectum, providing an entry point for HIV. 4. **Sharing Sex Toys:** If sex toys are shared and not cleaned or covered with a new condom between uses, they can potentially transmit HIV and other STIs. 5. **Drug Use During Sex:** Some people use drugs or alcohol before or during sex. This may lead to risky sexual behaviors, such as not using a condom, which increases the risk of HIV transmission. 6. **Commercial Sex Work:** Engaging in commercial sex work often involves a higher number of sexual partners and greater frequency of unprotected sex, which can increase the risk of HIV transmission.   To reduce the risk of HIV, it is recommended to always use a condom during sex, limit the number of sexual partners, get tested for HIV and other STIs regularly, and use Pre-exposure prophylaxis (PrEP) if you're at high risk of contracting HIV. In addition, never share needles or drug equipment. |  |
| 1. What is safer sex? | |
| Safer sex (also known as safe sex) refers to sexual activities that reduce the risk of transmitting or getting sexually transmitted infections (STIs), including HIV. While no sex is 100% safe, there are many ways to make sex safer.  Here are some strategies for safer sex:   1. **Use a Condom Correctly Every Time You Have Sex**: Condoms (both external/“male” and internal/“female” types) provide a barrier that can prevent the exchange of sexual fluids and reduce skin-to-skin contact, both of which can transmit STIs. 2. **Get Tested Regularly**: Both you and your partner(s) should get tested for STIs, including HIV, on a regular basis, and share your test results with each other. The frequency of testing should be based on your sexual activity and risk factors. Regular testing means that if you do get an STI, including HIV, you can get treatment promptly, which can improve your health, prolong your life, and greatly lower your chance of spreading the infection to others. 3. **Limit Your Number of Sexual Partners**: The more partners you have, the more likely you are to be exposed to STIs. Having sex with one partner who only has sex with you and who has been tested and is negative for STIs can lower your risk. 4. **Get Vaccinated**: Vaccines are available to prevent human papillomavirus (HPV) and hepatitis B, both of which can be transmitted through sexual activity. 5. **Use PrEP and PEP**: If you are at high risk of HIV, consider taking pre-exposure prophylaxis (PrEP), a daily pill that can prevent HIV infection. Post-exposure prophylaxis (PEP) is another medication that can be taken within 72 hours of possible exposure to HIV to prevent infection. 6. **Choose Lower-Risk Sexual Activities**: Oral sex is much less risky than vaginal or anal sex. Mutual masturbation is also a lower-risk option. 7. **If You Use Drugs, Don't Share Needles**: Sharing needles can transmit HIV and other viruses like hepatitis C. 8. **For HIV-Positive Individuals, Adhere to Treatment**: If you have HIV, it's crucial to take your medications as prescribed. Doing so can keep you healthy and prevent transmission to others. When effectively treated, people with HIV can achieve a viral load so low it's undetectable in a blood test, and at that point, the virus is not transmissible to sexual partners—a status referred to as "Undetectable equals Untransmittable," or "U=U".   Remember that what counts as 'safer' varies greatly depending on the individuals involved, their sexual practices, and their specific health circumstances. Always communicate openly with your partner(s) about safer sex and STI status, and consult with a healthcare provider for personalized advice. |  |
| 1. Which sexual practice has the highest risk of getting HIV? | |
| Unprotected anal intercourse has the highest risk of HIV transmission among sexual practices. This risk is especially high for the receptive partner (the person on the "bottom"), as the lining of the rectum is thin and may be more susceptible to tearing and allowing HIV to enter the body.  The risk of HIV transmission during unprotected anal intercourse is significantly higher than during unprotected vaginal intercourse. The insertive partner (the person on the "top") in anal intercourse is also at risk, but the risk is less than for the receptive partner.  Other sexual practices that carry a significant risk of HIV transmission include vaginal intercourse and sharing sex toys, especially if they are not cleaned between uses or used with a new condom each time.  Oral sex carries a lower risk, but it's still possible to transmit HIV this way, particularly if there are open sores, cuts, or abrasions in the mouth, or if ejaculation occurs in the mouth.  It's also important to note that the presence of other sexually transmitted infections (STIs) can increase a person's risk of acquiring or transmitting HIV.  Using condoms consistently and correctly, getting regularly tested for HIV, and taking pre-exposure prophylaxis (PrEP) if you're at high risk can significantly reduce the risk of HIV transmission. If a person with HIV is taking antiretroviral therapy (ART) as prescribed and has an undetectable viral load, they have effectively no risk of transmitting HIV to their HIV-negative partners. This is commonly referred to as "U=U" or "Undetectable equals Untransmittable". |  |
| 1. Who Is at Risk for HIV? | |
| While HIV can affect anyone, certain behaviors and situations can increase a person's risk of contracting the virus. Some of these risk factors include:   - Unprotected Sex: Having vaginal or anal sex without a condom with someone who is HIV-positive or whose HIV status is unknown increases the risk of HIV transmission. Receptive anal sex (bottoming) is the highest-risk sexual behavior for HIV transmission. - Multiple Sexual Partners: The more sexual partners you have, the more likely you are to have a partner with HIV whose virus is not well controlled, or to have a partner with a sexually transmitted infection, both of which increase the risk of HIV transmission. - Injection Drug Use: Sharing needles or syringes, rinse water, or other equipment (works) used to prepare drugs for injection with someone who has HIV. - Sexually Transmitted Infections (STIs): Having an STI can increase the risk of getting or transmitting HIV. - Blood Transfusion or Organ/Tissue Transplant: This is very rare in countries where rigorous testing is done.   Certain populations are disproportionately affected by HIV due to a range of social and economic factors. These populations can include men who have sex with men, transgender women, people of color, and people living in certain geographic areas.  Regardless of these risk factors, anyone who is sexually active or uses injection drugs can benefit from regular HIV testing. It's also important to consider preventive measures like using condoms consistently and correctly, taking pre-exposure prophylaxis (PrEP) if you're at high risk, and using clean needles and works if you inject drugs.  Always consult a healthcare provider for advice tailored to your specific circumstances and needs. |  |
| 1. Which group of people have higher risk of being HIV postiive? | |
| As of my knowledge cutoff in September 2021, certain populations are disproportionately affected by HIV/AIDS. These include:   - Men Who Have Sex with Men (MSM): Globally, this group has been significantly impacted by HIV. In many countries, including the United States, MSM make up the majority of new HIV diagnoses. - Transgender Women: Transgender women, particularly those of color, are at a disproportionately high risk for HIV infection. Factors contributing to this increased risk include social and economic marginalization, stigma, and lack of access to healthcare and HIV prevention services. - People Who Inject Drugs (PWID): Sharing needles, syringes, and other drug injection equipment increases the risk of HIV transmission. - Sex Workers: Due to the nature of their work, sex workers often face higher rates of unprotected sex, multiple sexual partners, and other risk factors that increase the chance of HIV transmission. Stigma and legal issues often prevent access to prevention, testing, and treatment services. - People in Correctional Facilities: Incarcerated populations have higher rates of HIV infection, often due to high-risk behaviors such as unprotected sex and drug use. - People of Color, particularly African Americans and Latinos in the United States: In the US, racial and ethnic minorities have been particularly hard hit by the HIV epidemic, largely due to structural and socioeconomic disparities. - People Living in Certain Geographic Regions: In certain parts of the world, including Sub-Saharan Africa and Eastern Europe/Central Asia, HIV rates are particularly high.   It's important to note that while these groups have higher rates of HIV infection, anyone can get HIV and everyone should take steps to protect themselves and others. It's also crucial to fight stigma and discrimination, which can discourage people from getting tested, accessing treatment, and disclosing their HIV status. Everyone has a role to play in the fight against HIV/AIDS.  Please consult the most current data from reliable health resources for updates beyond September 2021. |  |
| 1. Does HIV only affect MSM, sex workers and drug users? | |
| No, HIV does not only affect men who have sex with men (MSM), sex workers, and drug users. While these groups have been identified as high-risk populations due to specific behaviors and societal factors, anyone can contract HIV. The virus does not discriminate based on sexual orientation, profession, or lifestyle.  HIV is primarily transmitted through unprotected sexual intercourse (vaginal or anal), sharing injection drug equipment, and from mother to child during childbirth or breastfeeding. Less commonly, it can be transmitted through blood transfusions or organ transplants, though this is rare in countries where these products are tested for HIV.  Therefore, anyone who engages in these behaviors—regardless of whether they identify as MSM, are sex workers, or use injection drugs—is at risk. This includes heterosexual men and women, people in monogamous relationships, and people who do not use drugs.  Moreover, certain factors can increase the risk of HIV transmission, such as having another sexually transmitted infection, having multiple sexual partners, and living in a community where HIV prevalence is high.  It's important for everyone, regardless of their perceived risk, to understand how HIV is transmitted and how to protect themselves. This can include using condoms consistently and correctly, reducing the number of sexual partners, never sharing needles or other drug equipment, and getting tested regularly for HIV. For people at high risk, taking pre-exposure prophylaxis (PrEP) can also help prevent HIV.  Consult a healthcare provider for advice tailored to your specific circumstances and needs. |  |
| 1. Is there a cure or vaccine for HIV? | |
| As of my knowledge cutoff in September 2021, there is neither a definitive cure nor a vaccine for HIV.  However, while HIV/AIDS remains a chronic condition, it can be managed effectively with antiretroviral therapy (ART). ART involves taking a combination of HIV medicines every day to control the virus. Although it doesn't cure HIV, it can allow people with the virus to live long, healthy lives and greatly reduces the risk of HIV transmission.  The search for an HIV vaccine has been ongoing for many years, but so far, no vaccine has been approved for public use. Several potential vaccines are in various stages of testing, and there have been some promising results, but it's not yet clear when or if an effective vaccine will be available.  In recent years, there's also been significant interest in the possibility of curing HIV. A few cases of apparent HIV cures have been reported, such as the "Berlin Patient" and the "London Patient," who appear to have been cured of HIV following stem cell transplants for cancer treatment. However, these treatments are risky, complex, and not feasible for widespread use. Researchers are exploring other potential cure strategies, but these are still in early stages of development.  For the latest information on the status of HIV vaccines and cures, please consult resources that are updated regularly, such as the U.S. Department of Health & Human Services, the World Health Organization, or the Centers for Disease Control and Prevention. |  |
| 1. How old a person with HIV may live? | |
| As of my last update in September 2021, with effective treatment, people with HIV can live nearly as long as those without the virus.  The advent of antiretroviral therapy (ART) has transformed HIV from a deadly disease into a manageable chronic health condition. ART works by preventing the virus from replicating, which allows the immune system to recover and fight off infections and diseases.  A person who is diagnosed with HIV early in the course of infection, and who is linked to appropriate care and treatment, can have a nearly normal life expectancy. However, the life expectancy for someone with HIV can vary based on several factors, including their age at diagnosis, the stage of the disease at diagnosis, their overall health, their access to and consistency in taking ART, and their lifestyle factors such as smoking, substance use, and other concurrent health conditions.  It's important to note that although ART has dramatically improved life expectancy for people with HIV, it doesn't cure the disease. People with HIV must take HIV medicine every day for the rest of their lives (unless a cure is found), and there can be complications and side effects associated with long-term HIV treatment.  For the most current information on HIV life expectancy, you should refer to recent scientific studies and guidelines from authoritative health bodies such as the World Health Organization or the Centers for Disease Control and Prevention. |  |
| 1. What does it mean U=U? | |
| U=U stands for "Undetectable equals Untransmittable." This phrase is based on scientific research which found that people living with HIV who have an undetectable viral load thanks to effective treatment cannot transmit the virus to their sexual partners.  Here's what the terms mean:   - Undetectable: When a person living with HIV is on regular treatment, the amount of HIV in their body can be reduced to levels that are 'undetectable' by standard viral load tests. - Untransmittable: When the level of HIV is 'undetectable,' the virus cannot be transmitted to another person during sex, even if condoms are not used.   This principle underscores the importance of individuals living with HIV to start and adhere to treatment both for their own health and to prevent transmission to others.  However, it's essential to note that U=U applies only to HIV transmission. Even with an undetectable viral load, there is still the risk of getting or transmitting other sexually transmitted infections (STIs). Therefore, safer sex practices, such as using condoms, are still important to prevent other STIs. |  |
| 1. Why people with HIV are stigmatized? | |
| Stigmatization of individuals living with HIV is unfortunately common and can be attributed to a number of factors:   - Lack of Understanding: Many people do not fully understand how HIV is transmitted. This can lead to unnecessary fear and discrimination against those who are living with the virus. - Association with Sex and Drug Use: HIV is often associated with behaviors that some societies or cultures might find morally objectionable or illegal, such as unprotected sex, multiple sexual partners, homosexuality, or intravenous drug use. This can lead to moral judgments and stigmatization. - Fear of Death: Since HIV was once a terminal illness and is still life-threatening if untreated, fear can lead to discrimination. - Invisibility of the Illness: Because HIV is not immediately visible, people may treat someone living with HIV differently due to fear of catching the virus themselves. - Media Representation: The portrayal of people living with HIV in the media can influence societal attitudes. Negative or inaccurate portrayals can perpetuate stereotypes and stigma. - Prejudice Against Groups: HIV has disproportionately affected certain communities, including men who have sex with men, people who inject drugs, and sex workers. Preexisting prejudices against these groups can worsen HIV stigma.   HIV-related stigma and discrimination can have severe consequences, including discouraging individuals from getting tested, hindering access to treatment, affecting mental health, and contributing to the spread of the virus.  Public education about HIV transmission, treatment, and prevention, as well as laws and policies to protect the rights of people living with HIV, are important tools to fight against HIV stigma and discrimination. |  |
| 1. Should I declare my HIV positivity to my general practitioner? | |
| Yes, it is highly recommended to inform your general practitioner (GP) if you test positive for HIV. There are several reasons for this:  Care Coordination: Your GP can help coordinate your care, work with your HIV specialist, and ensure that any other health issues you have are managed effectively in conjunction with your HIV treatment.  Complete Health Picture: Sharing your HIV status with your GP provides them with a complete picture of your health. This is important for making decisions about your care, including prescribing medications that won't interfere with your HIV treatment.  Support and Resources: Your GP can provide support and resources, refer you to appropriate services such as counseling or support groups, and answer questions about living with HIV.  Remember, your GP is bound by confidentiality laws and cannot disclose your HIV status without your consent, except in very specific circumstances (like if there is an immediate threat to your health or the health of others).  Living with HIV means you'll need to take special care of your health, and your GP can be a vital part of your healthcare team. |  |
| 1. I have just discovered to have HIV, what could happen if I don’t take the pills? | |
| If you've been diagnosed with HIV and don't start antiretroviral therapy (ART), the virus will continue to multiply and attack your immune system. Over time, this can lead to the development of AIDS (Acquired Immune Deficiency Syndrome), which is the most advanced stage of HIV infection.  The risks and consequences of not taking ART include:   - Weakened Immune System: HIV attacks and destroys CD4 cells, which are crucial for a healthy immune response. Over time, this can significantly weaken your immune system and make you more susceptible to infections and diseases that your body would otherwise be able to fight off. - Opportunistic Infections and Cancers: A weakened immune system can lead to opportunistic infections, which are infections that occur more frequently or are more severe in people with weakened immune systems. You would also be at a higher risk for certain types of cancers. - Development of AIDS: If left untreated, HIV can progress to AIDS, a serious condition that can lead to severe weight loss, brain disorders, and ultimately, death. - Transmission to Others: Without treatment, the amount of HIV in your body (viral load) is likely to be high, which increases the risk of transmitting the virus to others. - Starting ART as soon as possible after diagnosis can help keep your immune system strong, control the virus, and prevent transmission to others. With proper treatment, people with HIV can live long, healthy lives.   Remember, it's crucial to consult with your healthcare provider to understand your specific situation and treatment options. This information is based on knowledge available up until my last training cut-off in September 2021, and there may have been advancements or changes in the understanding of HIV treatment since then. Always consult with a healthcare provider for the most current and personal advic. |  |
| 1. I have just discovered to have HIV; how long do I have to live? | |
| With the advances in antiretroviral therapy (ART), HIV has become a manageable chronic condition. Many people who are diagnosed with HIV early, start treatment promptly, and manage it effectively can have nearly the same life expectancy as those without the disease.  Treatment with ART suppresses the virus, even to undetectable levels in your blood, which not only helps to prevent progression to AIDS (the last stage of the HIV infection that occurs when your immune system is badly damaged) but also reduces the risk of transmission to others.  However, it's important to note that individual health outcomes can depend on a number of factors, including the age at diagnosis, overall health, lifestyle choices (like diet, exercise, and avoiding tobacco and alcohol), and timely and consistent access to high-quality healthcare.  If you've just received an HIV diagnosis, it's essential to seek care from a healthcare provider experienced in treating HIV as soon as possible. They can guide you on starting and adhering to treatment, managing any side effects, and dealing with other aspects of living with HIV, including emotional or mental health concerns.  Remember, being diagnosed with HIV is life-changing, but it's not a death sentence. Many people with HIV are leading active, fulfilling lives. Support is available, including from healthcare providers, support groups, and organizations dedicated to helping people with HIV. |  |
| 1. If I received oral sex, may I get HIV infection? | |
| HIV transmission from receiving oral sex (that is, having someone perform oral sex on you) is considered to be low risk. However, it's not zero. There are a few factors that could potentially increase the risk:   1. **Oral Health:** If the person performing oral sex has bleeding gums, mouth ulcers, or any other conditions that allow HIV in their blood or sexual fluids to enter their mouth, the virus could potentially be passed on. 2. **Sexual Health:** If you have an open sore or wound on your genitals, this could provide an entry point for HIV. 3. **STIs:** If either partner has an untreated sexually transmitted infection, this could increase the risk.   That said, compared to vaginal or anal sex, the risk of acquiring HIV through receiving oral sex is significantly lower. Nonetheless, safer sex practices should be adopted to minimize risks. You can further reduce the risk by using a condom or dental dam during oral sex, and by maintaining good oral health.  It's also worth noting that other sexually transmitted infections can be passed on more easily through oral sex, such as gonorrhea, syphilis, and herpes. Regular testing for HIV and other STIs is a good idea if you're sexually active, especially with new or multiple partners. |  |
| 1. May I get HIV infection with receptive anal sex? | |
| Yes, receptive anal sex (being the "bottom") is the sexual behavior that carries the highest risk of acquiring HIV. This is because the lining of the rectum is thin and may allow the virus to enter the body during intercourse.  The risk is much higher if your partner has HIV and is not on antiretroviral treatment, or if you or your partner has another sexually transmitted infection (STI). The risk is also higher if no condom is used or if the condom breaks or slips off.  However, there are ways to protect yourself:   - Condoms: Using condoms correctly and consistently every time you have sex can significantly reduce the risk of HIV and other STIs. - PrEP: Pre-exposure prophylaxis (PrEP) is a daily medication that can greatly reduce your risk of getting HIV. It is recommended for people at high risk of HIV, including those who have anal sex without condoms with partners of unknown or positive HIV status. - Partner's viral load: If your partner is living with HIV, their viral load can affect the risk of transmission. If they are on antiretroviral therapy and have an undetectable viral load, they cannot transmit HIV to you. This is summarized as "U=U" (Undetectable equals Untransmittable). - Regular testing: Regular testing for HIV and other STIs is important for everyone who is sexually active, especially if you have multiple partners or engage in sex without condoms.   Remember to always consult with a healthcare provider for advice tailored to your specific circumstances. |  |
| 1. May I get HIV infection with penetrative anal sex | |
| Yes, performing penetrative anal sex (being the "top") carries a risk of acquiring HIV, especially if it is unprotected (without a condom) and your partner is HIV-positive and not on antiretroviral therapy. Although the risk is lower for the insertive partner compared to the receptive partner (the "bottom"), there is still a risk, as HIV can enter the body through the urethra or through any small cuts, sores, or abrasions that may be on the penis.  To reduce the risk of acquiring HIV and other sexually transmitted infections (STIs) when engaging in anal sex, you can take several precautions:   - Use Condoms: Always use a condom during anal sex. Condoms create a barrier that helps prevent the spread of HIV and other STIs. It's also important to use a water-based or silicone-based lubricant, as this can help prevent condoms from breaking during anal sex. - PrEP: Pre-exposure prophylaxis (PrEP) is a daily medication that can significantly reduce the risk of acquiring HIV. It's recommended for individuals who are at high risk of acquiring HIV. - Regular Testing: Regularly test for HIV and other STIs. Knowing your status (and your partner's) can help you make informed decisions about the sexual activities you engage in. - Partner's Viral Load: If you're having sex with a partner who is HIV-positive, it's important to know their viral load. If they are on antiretroviral therapy and have an undetectable viral load, they cannot transmit HIV to you (known as "U=U", Undetectable equals Untransmittable).   Remember to always consult a healthcare provider for advice tailored to your specific circumstances and to discuss any questions or concerns you may have about HIV transmission and prevention. |  |
| 1. If a man receive oral sex, may he getting HIV infection? | |
| The risk of getting HIV by receiving oral sex (meaning that your penis is in someone else's mouth) is extremely low.  HIV is not easily transmitted through oral sex. The main risk would be if the person performing oral sex has bleeding gums, mouth sores, or a sexually transmitted infection (STI) in their throat, which could potentially allow the virus to enter their bloodstream. Even so, the risk is considered to be very low.  However, while the risk of HIV transmission through oral sex is low, other STIs such as gonorrhea, syphilis, and herpes can be transmitted this way. Using a condom or dental dam during oral sex can reduce the risk of these infections.  It's important to remember that regular testing for HIV and other STIs is a crucial part of sexual health, especially if you're sexually active with multiple partners or in a high-risk group. Always consult with a healthcare provider for personalized advice based on your specific circumstances and needs. |  |
| 1. If I do a blowjob, may I get HIV infection? | |
| Oral sex, meaning mouth-to-penis (fellatio), mouth-to-vagina (cunnilingus), or mouth-to-anus (anilingus) contact, generally carries a lower risk of HIV transmission than other sexual activities such as anal or vaginal sex. However, it is not entirely risk-free.  In the case of giving oral sex to a man (fellatio), the risk of acquiring HIV is generally low but can increase under certain conditions:   - Ejaculation in the mouth: If the man ejaculates and you have cuts or sores in your mouth or throat, or if you have inflammation caused by a sexually transmitted infection or recent dental work, the virus can potentially enter your bloodstream. - Oral health: If you have bleeding gums, ulcers, cuts, sores, or infections in the mouth, these could provide a route for HIV to enter the body. - The man's viral load: If the man is HIV-positive, the risk can depend on his viral load. If he's on antiretroviral therapy (ART) and his viral load is undetectable, he can't transmit HIV to you. This concept is referred to as U=U (Undetectable equals Untransmittable).   You can reduce your risk of getting HIV through oral sex by using barriers like condoms or dental dams, taking pre-exposure prophylaxis (PrEP) if you are at high risk, and ensuring both you and your partner are regularly tested for HIV and other STIs.  Remember, it's always important to consult with a healthcare provider for advice tailored to your specific circumstances. |  |
| 1. If I do a blowjob without ejaculation, may I get HIV infection? | |
| The risk of acquiring HIV from performing oral sex (a blowjob) on a man without ejaculation is generally considered to be low. HIV is primarily spread through direct contact with certain body fluids—blood, semen, pre-seminal fluid (pre-cum), rectal fluids, vaginal fluids, and breast milk—from a person who has HIV.  While pre-ejaculate, or pre-cum, can contain HIV, the concentration of HIV in pre-cum is generally less than in semen. Therefore, if a man does not ejaculate during oral sex, the risk of HIV transmission is likely lower.  However, even without ejaculation, it's not completely risk-free. If you have cuts, sores, or abrasions in your mouth or throat, or if you have oral infections or inflammation, these could potentially provide a route for the virus to enter your body, if the man is HIV-positive.  To minimize risk, you can use a condom during oral sex. If you're at high risk of getting HIV (for example, you have a sexual partner who is HIV-positive), you might also consider pre-exposure prophylaxis (PrEP), a daily medication that can help prevent HIV infection.  As always, regular testing for HIV and other sexually transmitted infections is an important part of maintaining sexual health. You should consult with a healthcare provider to discuss the best ways to protect yourself based on your specific circumstances. |  |
| 1. If I received a blowjob, may I get HIV infection? | |
| Receiving oral sex (fellatio) is considered to be a low risk activity for the transmission of HIV, but the risk is not zero. The person providing oral sex could potentially transmit the virus to the person receiving oral sex if the former has HIV and there are sores, cuts, or open wounds in their mouth, or if the latter has sores, wounds, or a sexually transmitted infection in the genital area.  It's worth noting that the risk of HIV transmission is higher if ejaculation occurs in the mouth, particularly if the person giving oral sex has poor oral health, gum disease, or open sores in their mouth.  However, the risk is significantly reduced if the HIV-positive partner is on antiretroviral therapy and has an undetectable viral load.  The best protection measures include getting regularly tested, knowing your partner's HIV status, and using barrier methods such as condoms. Although oral sex is considered lower risk, practicing safer sex is always advised. |  |
| 1. If I do cunnilingus, may I get HIV infection? | |
| While the risk of HIV transmission through oral sex is generally lower than other sexual activities like vaginal or anal intercourse, it's not zero. Cunnilingus (oral sex performed on a female) can theoretically transmit HIV if the person giving oral sex has sores, cuts, or open wounds in their mouth, or if the person receiving oral sex is menstruating, or has genital sores or other STDs. The risk increases if either partner has gum disease, oral cancer, or any other condition that makes the oral mucosa more permeable.  It's important to note, however, that this risk is significantly reduced if the HIV-positive partner is on antiretroviral therapy and has an undetectable viral load.  As with all sexual activity, practicing safer sex is important. This can include using dental dams during oral sex, regular STI testing, and using pre-exposure prophylaxis (PrEP) if you are at a higher risk for HIV.  Remember, while cunnilingus may pose a risk for HIV transmission, the risk is much lower compared to vaginal or anal intercourse. The CDC classifies the risk from oral sex as "low but not zero".  The best way to protect yourself is to be informed about your partner's sexual health, to get tested regularly, and to use protection where appropriate. |  |
| 1. If I received cunnilingus, may I get HIV infection? | |
| Receiving cunnilingus, which is oral sex performed on a woman, is generally considered a low-risk activity for HIV transmission, but it's not zero. If the person performing oral sex has HIV and has open cuts, sores, or gum disease in their mouth, there is a small chance they could transmit the virus. If the person receiving cunnilingus has a sexually transmitted infection (STI) or any open sores or cuts in the genital area, this could also increase the risk.  The risk of HIV transmission is significantly reduced if the HIV-positive partner is on antiretroviral therapy and has an undetectable viral load.  In order to protect yourself, it's important to know the HIV status of your sexual partner, get tested regularly for STIs, and consider the use of barrier methods such as dental dams during oral sex.  Remember, though the risk associated with cunnilingus is considered low, it's always important to engage in safer sex practices. |  |
| 1. It is possible for women who have sex with women getting HIV infection? | |
| Yes, women who have sex with women can get HIV. While the risk of transmission is generally lower than for some other forms of sexual activity, especially unprotected penetrative sex, it's not zero.  There are several ways that HIV could theoretically be transmitted between women, including:   - Sharing sex toys: If a sex toy is shared and has been in contact with the vaginal or anal fluids or blood of a woman with HIV, there is a risk of transmission, especially if the toy is used immediately by another person and is inserted deeply. - Finger/fist insertion: If a woman with HIV has cuts or sores on her hand and inserts her fingers or fist into another woman's vagina or anus, there could be a risk of transmission. - Oral sex: If a woman with HIV is menstruating and her partner performs oral sex, the partner could be at risk if they have sores in their mouth, bleeding gums, or are infected with a sexually transmitted infection (STI). - Other STIs: If a woman has another STI, it can increase the risk of HIV transmission. - Use of drugs during sex: If a woman uses intravenous drugs and shares needles with an HIV-positive person, she's at a high risk for contracting HIV.   Therefore, safer sex strategies such as the use of barriers (like dental dams or condoms on sex toys), regular testing for STIs including HIV, and avoiding the sharing of needles, can help reduce the risk of transmission between women. PrEP (pre-exposure prophylaxis) is also an option for people at high risk of contracting HIV, including women who have sex with women.  While the absolute risk is lower than for some other groups, it's still important for women who have sex with women to talk about safer sex, get tested regularly, and be aware of the various risk factors for HIV transmission. |  |
| 1. Which precaution may I take to reduce the risk of HIV transmission during a cunnilingus? | |
| Here are a few precautions you can take to reduce the risk of HIV transmission during cunnilingus:   - Use a Dental Dam: This is a thin square of latex that can be placed over the vulva to create a barrier between the mouth and the genitals. If a dental dam is not available, you can also use a cut-open latex condom. - Maintain Good Oral Hygiene: Brushing and flossing regularly can help keep your gums healthy and reduce the chance of bleeding, which can increase the risk of HIV transmission. - Avoid Oral Sex if There are Mouth or Genital Sores: Any kind of open sore or inflammation can increase the risk of HIV transmission. This includes cuts, sores, or abrasions in the mouth, as well as genital sores or infections. - Know Your Status and Your Partner's Status: Regularly testing for HIV and other sexually transmitted infections (STIs) is crucial. Be open and honest with your partner about your status and ensure they do the same. - Antiretroviral Therapy (ART): If you or your partner is HIV-positive, taking antiretroviral therapy (ART) can significantly reduce the risk of transmitting HIV. It's possible for an HIV-positive person on ART with an undetectable viral load to not transmit the virus to others. - Pre-exposure Prophylaxis (PrEP): This is a way for people who are at very high risk of getting HIV to prevent HIV infection by taking a pill every day. Talk to your healthcare provider to see if PrEP could be right for you.   Remember that no method is 100% effective at preventing HIV transmission, and the methods used should reflect the level of risk you are comfortable with. Always have open, honest discussions about sexual health with your partner. |  |
| 1. If I do scissoring with my partner, May I get HIV infection? | |
| "Scissoring" refers to a sexual act typically associated with lesbian couples where partners rub their vulvas together. The term is used to describe a variety of sexual activities involving genital-to-genital contact.  Regarding HIV transmission, the risk is generally lower compared to activities like vaginal or anal sex, but it's not zero. Theoretically, if one partner is HIV-positive and there is direct contact between mucous membranes or broken skin, there is a possibility of transmission. If one or both partners have sores or cuts, or if one partner is menstruating, the risk could be higher.  As with any sexual activity, safer sex strategies can reduce risk. These strategies include regular testing for sexually transmitted infections (STIs), including HIV, and being aware of any cuts, sores, or menstrual blood that might increase the risk of transmission. If one partner is known to be HIV-positive, antiretroviral therapy (ART) to maintain an undetectable viral load can also significantly reduce the risk of transmission.  Please consult with your healthcare provider for personalized advice about STI and HIV prevention. |  |
| 1. If I received fisting, may I get HIV infection? | |
| Fisting refers to a sexual practice where an entire hand or fist is inserted into a partner's vagina or anus.  The act of fisting can potentially lead to HIV transmission if precautions are not taken. If the person performing the fisting has open cuts, sores, or wounds on their hands and the person receiving has tears or cuts in the vagina or anus, HIV could potentially be transmitted, especially if the person with the wounds or cuts is HIV-positive.  Moreover, because fisting can cause tears in the rectal or vaginal tissue (even small ones that you might not see or feel), it may increase the likelihood of HIV transmission during subsequent sexual activity if condoms or other barrier methods are not used.  Here are some ways to reduce the risk:   - Use Gloves: Latex or nitrile gloves can act as a barrier to prevent the transmission of HIV and other sexually transmitted infections (STIs). They can also help to prevent causing any damage to the rectal or vaginal tissues. - Use Lube: A water-based or silicone-based lubricant can reduce the risk of tears or damage to the rectum or vagina. - Go Slowly and Carefully: This can help prevent causing any damage or tears to the rectum or vagina. - Know Your Status and Your Partner's Status: Regularly testing for HIV and other STIs is crucial. Be open and honest with your partner about your status and ensure they do the same. - Antiretroviral Therapy (ART): If you or your partner is HIV-positive, taking antiretroviral therapy (ART) can significantly reduce the risk of transmitting HIV.   Remember, while these measures can help reduce the risk, they cannot eliminate it entirely. Always consult with a healthcare provider for personalized advice about STI and HIV prevention. | |
| 1. If I received rimming, may I get HIV infection? | |
| Receiving anilingus, or having someone perform oral sex on your anus ("rimming"), is generally considered a low-risk activity for acquiring HIV. The lining of the mouth is quite resistant to HIV, and the virus does not survive well in saliva.  However, while the risk of HIV transmission is low, other sexually transmitted infections (STIs) such as herpes, gonorrhea, and syphilis can be more easily transmitted through this activity. If the person performing anilingus has oral sores, bleeding gums, or an STI in their throat, these factors could potentially increase the risk.  Furthermore, it's possible for bacteria in fecal matter to cause health issues, such as hepatitis A or intestinal parasites.  To reduce your risk, you could use a dental dam or a cut-open condom to provide a barrier during anilingus. It's also important to maintain good hygiene and get regularly tested for HIV and other STIs if you're sexually active. |  |
| 1. If I do rimming, may I get HIV infection? | |
| The risk of getting HIV from performing anilingus, or oral-anal sex (also known as "rimming"), is low. HIV is not easily transmitted through oral routes, and the saliva has enzymes that inhibit the virus.  However, there are other health risks associated with performing anilingus:  Other STIs: You can be more susceptible to other sexually transmitted infections such as herpes, syphilis, or gonorrhea, which can be transmitted through skin-to-skin contact or contact with fecal matter.  Hepatitis A and B: These can be contracted through contact with fecal matter.  Parasitic Infections: Parasites like Giardia or amoebas can be present in fecal matter and can cause gastrointestinal illness.  To reduce these risks, a barrier method should be used, such as a dental dam or a cut-open condom, during anilingus. Good oral and personal hygiene is also important. If you're sexually active, especially with multiple partners, it's important to get regularly tested for HIV and other STIs. Also, vaccinations for Hepatitis A and B are recommended. |  |
| 1. Is it possible to get HIV infection by sharing sex toys? | |
| Yes, it is possible, although the risk of HIV transmission through shared sex toys is considered to be lower compared to other sexual behaviors. If a sex toy is used by someone with HIV and then immediately used by another person without being properly cleaned, the HIV virus could potentially be transmitted.  The risk increases if the toy is used for anal penetration and then used for vaginal penetration, or used by a person with HIV and then by a person without HIV. This is because HIV can live in certain body fluids (blood, semen, rectal fluids, vaginal fluids, and breast milk) and the virus could potentially be on the toy.  To reduce the risk of HIV transmission, as well as other sexually transmitted infections, follow these guidelines:   1. **Do not share sex toys**, or if you must share them, 2. **Use a new condom** on the toy when each new person uses it. 3. **Clean sex toys properly** between uses and between anal and vaginal use.   Also, get regularly tested for HIV and other STIs if you're sexually active, especially with multiple partners. Using Pre-Exposure Prophylaxis (PrEP) medication can also help reduce the risk of HIV infection in people who are at high risk. |  |
| 1. May I get HIV infection by drinking urine? | |
| Drinking urine is not a common route of HIV transmission. The virus is not present in urine in large enough amounts to infect another person. The main body fluids known to transmit HIV are blood, semen, pre-seminal fluids, rectal fluids, vaginal fluids, and breast milk. |  |
| 1. May I get HIV infection with a golden shower? | |
| The act of giving or receiving a "golden shower" (urinating on a sexual partner) is not generally considered a high-risk activity for HIV transmission. This is because urine is not one of the fluids that carry a high concentration of HIV. The primary fluids that can transmit HIV are blood, semen, pre-seminal fluids, rectal fluids, vaginal fluids, and breast milk.  However, if the person urinating has other sexually transmitted infections (STIs), there could be a risk if the urine comes into contact with mucous membranes or any open cuts or sores. Also, if there is any blood present in the urine of a person who has HIV, that could potentially increase the risk, although this scenario is relatively rare.  As always, it's important to use barrier methods for any sexual activity that could involve an exchange of body fluids, get regularly tested for HIV and other STIs if you're sexually active, and discuss sexual health with your partner(s). |  |
| 1. May I get HIV infection with squirting? | |
| "Squirting" refers to the act of expelling clear fluid from the urethra during sexual activity. While it's still a topic of some debate, it's generally agreed that the fluid involved in squirting is different from urine and likely comes from the Skene's glands, which are located near the female urethra and are sometimes referred to as the "female prostate."  The risk of HIV transmission from squirting would likely be low, because HIV is primarily spread through blood, semen, pre-seminal fluids, rectal fluids, vaginal fluids, and breast milk. However, if the fluid expelled during squirting was mixed with HIV-infected vaginal fluid or menstrual blood, there could potentially be a risk of HIV transmission.  If you're engaging in sexual activity with someone who is HIV positive or whose HIV status is unknown, the best way to reduce the risk of HIV transmission is by using a barrier method like a dental dam or condom, getting regularly tested for HIV and other sexually transmitted infections (STIs), and considering the use of Pre-Exposure Prophylaxis (PrEP) if you are at a high risk of HIV infection. Always consult with a healthcare professional for advice tailored to your specific circumstances. |  |
| 1. May I get HIV infection receiving masturbation? | |
| The risk of acquiring HIV through receiving masturbation (someone else masturbating you) is extremely low. HIV is transmitted through certain body fluids: blood, semen, pre-seminal fluids, rectal fluids, vaginal fluids, and breast milk. In the context of sexual activity, it's usually transmitted through unprotected sexual intercourse (vaginal or anal) or, less commonly, oral sex.  Masturbation generally does not involve an exchange of these body fluids. However, if the person masturbating you has cuts or sores on their hand and you have open cuts or sores on your genitals, there could potentially be a risk, although it would be very low.  To minimize the risk of HIV and other sexually transmitted infections (STIs), you should consider the following:   - Use a condom or other barrier method during any sexual activity that could involve an exchange of body fluids. - Get regularly tested for HIV and other STIs, especially if you have multiple sexual partners or are in a relationship with someone who has HIV. - Consider taking pre-exposure prophylaxis (PrEP) if you are at high risk for HIV.   As always, it's important to have open, honest conversations about sexual health with your sexual partner(s). |  |
| 1. May I get HIV infection if I masturbate a woman? | |
| The risk of acquiring HIV through manual stimulation or masturbation of a woman (using your hand to stimulate a woman's genitals) is extremely low. HIV transmission requires contact with certain bodily fluids such as blood, semen, vaginal fluids, or breast milk from a person who has HIV, and these fluids must come into contact with a mucous membrane or damaged tissue or be directly injected into your bloodstream (from a needle or syringe) for transmission to occur.  However, if you have cuts, sores, or abrasions on your hand, and you come into direct contact with vaginal fluids or menstrual blood from a woman who has HIV, there could theoretically be a risk of transmission. But overall, this risk is considered very low.  While the risk of HIV transmission through this activity is extremely low, other sexually transmitted infections (STIs) can be passed in this way, including herpes and HPV. So it's important to get regularly tested if you're sexually active, especially with multiple partners.  As always, it's important to have open, honest discussions with your sexual partners about HIV and STI status, prevention strategies, and testing. Please consult with a healthcare provider for personalized advice based on your specific circumstances. |  |
| 1. May I get HIV infection if I masturbate a man? | |
| The risk of acquiring HIV from masturbating a man (touching a man's genitals with your hands) is extremely low. HIV is transmitted through certain bodily fluids such as blood, semen, vaginal fluids, rectal fluids, or breast milk, and these fluids need to come into contact with a mucous membrane (found inside the rectum, vagina, penis, and mouth) or damaged tissue, or be directly injected into the bloodstream (via shared needles or syringes) for transmission to occur.  However, if you have cuts, sores, or abrasions on your hand and you come into direct contact with semen from a man who has HIV, there could theoretically be a risk of transmission. But again, the risk from this activity is considered extremely low.  Remember that while the risk of HIV transmission in this way is extremely low, other sexually transmitted infections (STIs) can be transmitted through sexual activity that doesn't involve intercourse, such as herpes and human papillomavirus (HPV).  Regular testing for HIV and other STIs is an important part of maintaining sexual health, especially if you're sexually active with multiple partners. Always consult with a healthcare provider for personalized advice based on your specific circumstances and needs. |  |
| 1. May I get HIV infection receiving a prostate massage? | |
| The risk of getting HIV from receiving a prostate massage is generally considered to be low. This is because HIV is transmitted through certain body fluids—blood, semen, pre-seminal fluids, rectal fluids, vaginal fluids, and breast milk—and a prostate massage usually does not involve an exchange of these fluids.  However, there could be a risk if the person performing the massage has HIV and there is direct contact between their bodily fluids (e.g., blood from a cut on their hand) and mucous membranes or cuts, sores, or abrasions in the anus or rectum.  Even so, the risk is generally considered to be much lower than with unprotected sexual intercourse or sharing needles or syringes.  To reduce risk:   - Ensure that any instruments used for the massage are cleaned and sanitized properly before and after use. - Consider using gloves and a water-based lubricant to prevent potential damage to the rectal lining. - Get tested regularly for HIV and other STIs if you are sexually active.   If you or your partner are at a high risk of HIV, you may want to discuss additional preventive measures with your healthcare provider, such as pre-exposure prophylaxis (PrEP). |  |
| 1. If I get pegged by my partner, are we at risk of getting HIV? | |
| "Pegging" refers to the act of a woman performing anal sex on a man using a strap-on dildo. The risk of HIV transmission through pegging is generally low, especially if the couple is monogamous and both partners are known to be HIV-negative. However, if there is any uncertainty about the HIV status of either partner, precautions should be taken.  While the woman (the partner doing the pegging) is not at significant risk of HIV transmission, the man (the partner being pegged) could potentially be at risk if his partner is HIV-positive and if the dildo used for pegging is not properly cleaned or covered with a new condom between uses.  If the woman has cuts or open sores on her genitals that come into contact with the dildo, and if the man has cuts or open sores in his rectum, there is a potential, albeit low, risk for HIV transmission if the woman is HIV-positive.  The best way to reduce the risk of HIV transmission during pegging is to:  Use a new condom on the dildo every time you switch from one partner to another, or from one type of sex (such as vaginal sex) to another (such as anal sex).  Clean the dildo properly before and after each use.  Use plenty of water-based lubricant to reduce the risk of tearing the skin in the rectum.  Get tested regularly for HIV and other sexually transmitted infections, and know your partner's HIV status.  Consider taking PrEP (pre-exposure prophylaxis) if you or your partner is at high risk for HIV.  Remember, although pegging has a relatively low risk of HIV transmission compared to other sexual behaviors, it can still potentially transmit other sexually transmitted infections (STIs), especially if precautions are not taken. Therefore, it's essential to use condoms and get regular STI testing. | |
| 1. Are there some Kink sexual practices at risk of getting HIV? | |
| Yes, some kink or BDSM (Bondage, Discipline/Dominance, Submission/Sadism, and Masochism) practices can potentially increase the risk of transmitting HIV, mainly when they involve sexual activities or sharing items that can lead to the exchange of body fluids or cause breaks in the skin.  Here are some specific ways that kink or BDSM practices can increase the risk of HIV transmission:   - Sexual Intercourse: Any sexual activity that involves vaginal or anal penetration can potentially transmit HIV, especially without a condom. This risk increases if either partner has another sexually transmitted infection or if the receptive partner is participating in receptive anal sex. - Sharing Sex Toys: HIV can be transmitted through sharing sex toys that are not cleaned properly between uses or used with a new condom each time. The risk increases if these toys cause small cuts or tears in the vagina, anus, or mouth. - Blood Play: This refers to BDSM practices that involve drawing blood, such as with needles or knives. If equipment is shared between people and not sterilized properly, it could potentially transmit HIV. - Fisting: This act can cause tears in the anus or vagina, potentially providing a route for HIV transmission, especially if no gloves are used or if they aren't changed between partners.   Remember, any sexual practice that can cause cuts, tears, or other breaks in the skin or mucous membranes can potentially increase the risk of HIV transmission. Also, sharing equipment that comes into contact with blood or other bodily fluids can increase risk. To minimize this risk, always use condoms and/or dental dams, don't share sex toys without cleaning them or changing the condom on them, wear latex gloves for any activities that might cause bleeding, and consider taking PrEP if you are at high risk for HIV. Always negotiate and agree upon safe words and boundaries to ensure all activities are consensual and safe.  Lastly, get regularly tested for HIV and other sexually transmitted infections, and seek medical attention if you have any symptoms or concerns about potential exposure to HIV. |  |
| 1. What is the risk of transmitting HIV through kissing or deep kissing? | |
| The risk of transmitting HIV through kissing, including deep or 'French' kissing, is extremely low to nonexistent. HIV is not spread through saliva.  For transmission to occur, the virus must come into contact with certain types of body fluids—blood, semen, pre-seminal fluids, rectal fluids, vaginal fluids, and breast milk—and be able to enter the body through an appropriate entry site, such as the inside of the vagina or rectum, the opening at the tip of the penis, or cuts or sores.  While it's theoretically possible that HIV could be passed through deep kissing if both partners have large open sores in their mouth or bleeding gums, this is considered such a low risk that it's almost negligible.  However, other sexually transmitted infections (STIs) can be transmitted through oral contact, so it's always a good idea to maintain good oral hygiene and get regular check-ups if you're sexually active. |  |
| 1. May I get HIV infection from petting? | |
| "Petting" generally refers to sexual activity that doesn't involve penetration, such as touching, stroking, or kissing. The risk of getting HIV from petting is extremely low, as HIV is primarily transmitted through certain body fluids—blood, semen, pre-seminal fluids, rectal fluids, vaginal fluids, and breast milk. These fluids must come into contact with a mucous membrane or damaged tissue or be directly injected into the bloodstream for transmission to occur.  While petting can sometimes involve contact with these fluids, the risk of transmission is much lower than with activities such as unprotected vaginal or anal sex. However, if you or your partner have open cuts or sores, it is possible that there might be some risk, albeit still very low.  To protect yourself from HIV, it's important to use a barrier method, such as a condom, during any sexual activity that could involve an exchange of body fluids. Regular testing for HIV and other STIs is also important if you're sexually active, especially if you have multiple sexual partners or are in a relationship with someone who has HIV. If you are at high risk of HIV, you may want to discuss additional preventive measures with your healthcare provider, such as pre-exposure prophylaxis (PrEP). |  |
| 1. My partner has HIV infection, am I at risk of getting HIV if we do unprotected sex? | |
| Yes, if your partner has HIV and you are engaging in unprotected sex, you are at risk of getting HIV. HIV is transmitted through certain body fluids—blood, semen, pre-seminal fluid (pre-cum), rectal fluids, vaginal fluids, and breast milk—from a person who has HIV. These fluids must come in contact with a mucous membrane or damaged tissue or be directly injected into your bloodstream (from a needle or syringe) for transmission to occur. Mucous membranes are found inside the rectum, vagina, penis, and mouth.  However, there are ways to reduce your risk:  Condoms: Use a condom every time you have sex. When used correctly, condoms are highly effective in preventing HIV and other sexually transmitted infections (STIs).  PrEP: Pre-exposure prophylaxis (PrEP) is a medication that, when taken consistently, can significantly reduce your risk of getting HIV. It's recommended for people at high risk of getting HIV, including people who have a sexual partner who is HIV-positive.  Partner's viral load: If your partner is on antiretroviral therapy (ART) for HIV and has an undetectable viral load, they cannot transmit HIV to you. This is known as "U=U" (Undetectable equals Untransmittable). However, your partner must take the medication exactly as prescribed and get regular check-ups to ensure the viral load remains undetectable.  Regular testing: Regular testing for HIV and other STIs is important. Knowing your status (and your partner's) can help you make informed decisions about the sexual activities you engage in.  Remember, always consult with a healthcare provider for advice tailored to your specific circumstances. |  |
| 1. Does chemsex increase the risk of HIV infection? | |
| Yes, engaging in chemsex, which refers to using drugs like mephedrone, GHB/GBL, or crystal meth before or during sex, can significantly increase the risk of HIV transmission as well as other sexually transmitted infections (STIs).  There are several reasons for this:   - Increased Risky Behavior: Some drugs can lower inhibitions, leading to riskier sexual behavior such as having unprotected sex, multiple sexual partners, or more prolonged sex sessions, which can increase the chances of contracting HIV and other STIs. - Damage to Body Tissues: Some of these substances can cause damage to the rectal and vaginal tissues, making it easier for HIV and other STIs to enter the body. - Injection Drug Use: If these drugs are being injected and shared needles or syringes are used, this can directly transmit HIV. - Neglect of Regular Medication: Engaging in chemsex can sometimes lead to forgetting or neglecting to take regular medications, like pre-exposure prophylaxis (PrEP) for HIV prevention or antiretroviral therapy (ART) for those already living with HIV, which can increase HIV transmission risk. - It's essential to understand the risks associated with chemsex and to take steps to protect yourself, such as using condoms and getting regularly tested for HIV and other STIs. PrEP can also be considered as part of an HIV prevention strategy, especially for those engaging in higher-risk activities.   Remember, healthcare professionals are there to provide non-judgmental advice and support to help you protect your health. As of my last update in September 2021, it's highly advisable to consult with a healthcare provider about safe practices and reducing risk. Newer information may be available if you're reading this after my training cut-off. Always consult with a healthcare provider for the most current and personal advice. | |
| 1. Does circumcision increase the risk of HIV infection? | |
| Actually, circumcision has been found to decrease the risk of a man acquiring HIV from a female partner during vaginal sex. Multiple studies, including randomized controlled trials, have shown that circumcision can reduce a man's risk of acquiring HIV by up to 60%. This is thought to be because the foreskin has a high concentration of cells that the HIV virus can easily infect.  However, it's important to note that while circumcision can reduce the risk of HIV transmission, it does not eliminate it. Men who are circumcised can still acquire and transmit HIV, and they should continue to use other prevention methods such as condoms, PrEP if appropriate, regular HIV testing, and reduction of risky behaviors.  Also, it's important to remember that circumcision has not been proven to reduce the risk of men acquiring HIV during sex with men, or of women acquiring HIV during sex with men.  Please keep in mind that this information is based on knowledge available as of my last training cut-off in September 2021. Always consult with a healthcare provider for the most current and personal advice. | |
| 1. My partner has HIV infection with an undetectable viral load, am I at risk of getting HIV if we do unprotected sex? | |
| If your partner is living with HIV but has an undetectable viral load due to effective antiretroviral therapy (ART), the risk of sexual transmission of HIV to you is effectively zero. This concept is referred to as U=U, which stands for "Undetectable equals Untransmittable."  To maintain an undetectable viral load, your partner must take their HIV medication exactly as prescribed and attend all their medical appointments. Regular monitoring of their viral load is important to ensure that it remains undetectable.  However, it's important to note that while having an undetectable viral load prevents HIV transmission, it does not protect you or your partner from other sexually transmitted infections (STIs) like gonorrhea, syphilis, or chlamydia. Condoms can reduce the risk of other STIs.  As always, have regular check-ups and consultations with your healthcare provider to ensure you are taking the necessary precautions for your health. The decision to stop using condoms should be made with careful consideration and medical guidance. |  |
| 1. Can I get HIV from blood transfusions? | |
| The risk of contracting HIV from a blood transfusion is extremely low in countries where the blood supply is routinely screened for HIV. This screening process ensures that donated blood does not contain the virus before it is used in any medical procedure, including transfusions.  Since the mid-1980s, when testing became available, blood banks in many countries began screening all donated blood for HIV. This has greatly reduced the risk of HIV transmission through blood transfusions. For example, in the United States, the risk is estimated to be less than 1 in 1.5 million.  However, in some parts of the world where blood screening procedures may not be as robust, the risk could be higher.  Remember, this information is based on data up to my last training in September 2021, and there may have been advancements or changes in the field since then. Always consult with a healthcare provider for the most current and personal advice. |  |
| 1. May I get HIV infection sharing towels? | |
| No, HIV cannot be transmitted by sharing towels.  The virus is transmitted through certain body fluids—blood, semen, pre-seminal fluids, rectal fluids, vaginal fluids, and breast milk. The virus cannot survive long outside the human body, so it cannot be transmitted through objects such as toilet seats, towels, or bed sheets.  It's also worth noting that HIV cannot be transmitted through saliva, tears, or sweat. The virus is not spread through casual contact such as shaking hands, hugging, or sharing dishes or towels.  To protect yourself from HIV, it's important to use a condom during sex, never share needles, and get tested regularly if you're at risk. If you or your partner are at high risk of HIV, you may want to discuss additional preventive measures with your healthcare provider, such as pre-exposure prophylaxis (PrEP). |  |
| 1. May I get HIV infection by sharing a nail cutter? | |
| In theory, sharing a nail cutter could potentially transmit HIV if it has been contaminated with the blood of a person who has HIV and is then used by another person who gets cut by the same nail cutter. However, the risk is considered to be extremely low.  HIV does not survive well outside the body. This means that the virus on a contaminated surface, such as a nail cutter, would need to be fairly fresh and then directly access the bloodstream of the next person using the nail cutter. This set of circumstances makes the likelihood of transmission in this way very low.  It's always good hygiene practice not to share personal items that could become contaminated with blood, such as toothbrushes, razors, or nail cutters, not only due to HIV, but also because of the risk of transmitting other diseases such as hepatitis B and C.  To protect yourself from HIV, use a condom during sex, never share needles, get tested regularly if you're sexually active or at risk, and consider pre-exposure prophylaxis (PrEP) if you're at high risk of HIV. Consult with a healthcare professional for advice tailored to your specific circumstances. |  |
| 1. May I get HIV infection sharing a razor? | |
| While it's not considered a common way to transmit HIV, there is a small risk associated with sharing razors. This is because razors can cause small cuts and may come into contact with blood. If a razor was used by a person living with HIV and their blood is on the razor, the virus could potentially be transmitted to another person if they also cut themselves with the same razor. However, it's important to note that HIV is a fragile virus and doesn't survive well outside the body, so the risk of transmission this way is considered low.  Regardless, it's a good idea not to share razors or other personal items that can come into contact with blood, such as toothbrushes or nail clippers, to avoid potential exposure to bloodborne pathogens, including but not limited to HIV.  As always, if you have concerns about your risk of HIV transmission, it's best to speak with a healthcare provider who can provide advice based on your specific circumstances. Regular testing for HIV and other sexually transmitted infections is an important part of maintaining sexual health, particularly if you're sexually active with multiple partners. |  |
| 1. May I get HIV infection sharing a glass? | |
| No, you cannot get HIV from sharing a glass. HIV is not spread through casual contact or through sharing objects such as dishes, glasses, or food.  HIV is transmitted through certain body fluids—blood, semen, pre-seminal fluids, rectal fluids, vaginal fluids, and breast milk. These fluids must come into contact with a mucous membrane or damaged tissue, or be directly injected into the bloodstream (from a needle or syringe) for transmission to occur.  It's important to note that HIV does not survive well outside the body. This means that the virus on a contaminated surface would not be able to infect another person. Plus, HIV is not spread through saliva, so sharing a glass or other drinking vessel does not pose a risk.  To protect yourself from HIV, it's important to use a condom during sex, never share needles, and get tested regularly if you're at risk. If you or your partner are at high risk of HIV, you may want to discuss additional preventive measures with your healthcare provider, such as pre-exposure prophylaxis (PrEP). |  |
| 1. May I get HIV infection sharing a toothbrush? | |
| The risk of acquiring HIV by sharing a toothbrush is considered very low, but it's theoretically possible under certain circumstances. If the person living with HIV has blood in their mouth or on their toothbrush, and you also have gum disease, bleeding gums, sores, or cuts in your mouth, there could be a potential route for the virus to enter your bloodstream.  However, HIV is a fragile virus and does not survive well outside of the body. Once exposed to air, the amount of HIV in the blood starts decreasing quickly, reducing the risk of transmission. In the real-world, HIV transmission through sharing a toothbrush is extremely unlikely and there are no documented cases of this happening.  Despite this, it is generally not recommended to share toothbrushes as it can lead to the transmission of other diseases and bacteria. It's always a good idea to use your own personal care items and avoid sharing those that come into contact with blood, including toothbrushes and razors.  As always, if you have concerns about potential exposure to HIV, it's best to speak with a healthcare provider. Regular testing for HIV and other sexually transmitted infections is an important part of maintaining your sexual health. |  |
| 1. May I get HIV infection sharing cutlery? | |
| No, you cannot get HIV from sharing cutlery. HIV is not transmitted through casual contact or through sharing objects such as dishes, utensils, or food.  HIV is transmitted through certain body fluids—blood, semen, pre-seminal fluids, rectal fluids, vaginal fluids, and breast milk. These fluids must come into contact with a mucous membrane or damaged tissue, or be directly injected into the bloodstream (from a needle or syringe) for transmission to occur.  It's important to note that HIV does not survive well outside the body. This means that the virus on a contaminated surface would not be able to infect another person. Plus, HIV is not spread through saliva, so sharing cutlery or dishes does not pose a risk.  To protect yourself from HIV, it's important to use a condom during sex, never share needles, and get tested regularly if you're at risk. If you or your partner are at high risk of HIV, you may want to discuss additional preventive measures with your healthcare provider, such as pre-exposure prophylaxis (PrEP). |  |
| 1. I live with a person with HIV, which precautions should I take? | |
| Living with a person who has HIV requires understanding but not fear. HIV is not transmitted by casual contact, so you do not need to worry about contracting HIV from sharing space, casual touch, hugging, using the same dishes, or even kissing.  Here are some precautions you should consider:   - Avoid Contact with Blood: HIV is transmitted through certain body fluids, including blood. If the person living with HIV has a cut or wound, they should cover it with a bandage. If you are providing care, like helping with a bandage, use disposable gloves. - Do not Share Items that Can have Blood: Do not share toothbrushes, razors, or other personal items that can have blood on them. - Sexual Activity: If you are in a sexual relationship with the person living with HIV, consider the following: - Use condoms: Condoms are highly effective at preventing the sexual transmission of HIV when used correctly and consistently. - Consider PrEP: If you're HIV-negative and in an ongoing sexual relationship with an HIV-positive partner, pre-exposure prophylaxis (PrEP) could be an option. PrEP involves taking a specific HIV medication every day to reduce your risk of infection. - Undetectable = Untransmittable (U=U): If your partner is on HIV treatment and their viral load is undetectable, they cannot transmit HIV to you. - Cleanliness: Regularly clean shared spaces and wash shared linens, but remember, HIV does not survive long outside the body, and it cannot be transmitted through the environment. - Seek Medical Advice: If you have any doubts or questions, seek advice from a healthcare provider who understands HIV.   Remember, HIV is not transmitted through casual contact, so you don't need to fear living with, touching, or hugging someone who is living with HIV. Compassion and understanding are essential. |  |
| 1. May I get HIV infection from sitting in a public bathroom? | |
| No, you cannot get HIV from sitting on a toilet seat in a public bathroom. HIV cannot survive long outside the human body and it cannot reproduce outside a human host. It is not spread by:   - Air or water - Insects, including mosquitoes or ticks - Saliva, tears, or sweat that is not mixed with the blood of an HIV-positive person - Toilet seats or other shared facilities   HIV is transmitted through certain body fluids—blood, semen, pre-seminal fluid (pre-cum), rectal fluids, vaginal fluids, and breast milk—from a person who has HIV. These fluids must come in contact with a mucous membrane or damaged tissue or be directly injected into your bloodstream (from a needle or syringe) for transmission to occur.  If you have concerns about your risk of HIV transmission, it's best to speak with a healthcare provider who can provide advice based on your specific circumstances. Regular testing for HIV and other sexually transmitted infections is an important part of maintaining sexual health. |  |
| 1. May I get HIV infection from a mosquito bite? | |
| No, you cannot get HIV from a mosquito bite.  HIV is a human virus that does not replicate or survive well in insects. When a mosquito bites a person, it does not inject the blood of the last person it bit into the next person. Instead, the mosquito injects its own saliva, which acts as an anticoagulant to keep the blood from clotting while the mosquito is feeding.  Even if a mosquito did draw blood from a person with HIV, the virus would not survive inside the mosquito. The mosquito's digestive system breaks down the virus and it cannot reproduce within the mosquito, making it unable to transmit HIV.  This is why, despite mosquitoes being vectors for many diseases like malaria and Zika virus, they do not transmit HIV.  The primary ways HIV is transmitted include sexual contact, sharing of needles or syringes, exposure to the blood of someone living with HIV, and from mother to child during birth or breastfeeding. If you have concerns about HIV transmission, it's best to speak with a healthcare provider for accurate information. |  |
| 1. What is the risk of transmitting HIV through body piercing or tattooing? | |
| There is a potential risk of transmitting HIV and other blood-borne infections such as hepatitis B and C during body piercing or tattooing procedures if the equipment used has been contaminated with the blood of a person who has the virus and if proper sterilization methods are not followed.  The risk increases if the instruments used are not new or sterilized, or if the person doing the procedure is untrained and doesn't follow infection control procedures. This includes using new disposable gloves and new needles, and properly cleaning and disinfecting any surfaces or equipment that could come into contact with blood or body fluids.  However, it's important to note that in many countries, professional tattoo parlors and body piercing shops are required to follow strict hygiene and sterilization standards to minimize the risk of any infection, including HIV. These practices make the risk of transmission in these settings extremely low.  If you're considering getting a tattoo or body piercing, it's important to make sure that you choose a reputable professional who follows all required safety protocols. This includes using new or thoroughly sterilized equipment for each customer and following other good hygiene practices.  If you're in a location where professional standards aren't well enforced, it would be safer to avoid these procedures. This might be particularly relevant when travelling in regions where professional health and safety regulations aren't as rigorous. |  |
| 1. Does the birth control pill protect me to get HIV infection? | |
| No, birth control pills do not protect against HIV or other sexually transmitted infections (STIs). Birth control pills are a form of contraception designed to prevent pregnancy, not STIs or HIV.  HIV is transmitted through certain body fluids—blood, semen, pre-seminal fluids, rectal fluids, vaginal fluids, and breast milk—of a person who has HIV. Any sexual activity that involves an exchange of these fluids can result in transmission of the virus.  To protect against HIV, it's important to use a condom correctly and consistently every time you have sex. Regular testing for HIV and other STIs is also important if you're sexually active. If you're at high risk of HIV, you may want to consider pre-exposure prophylaxis (PrEP), a medication that can significantly reduce your risk of HIV infection.  Remember, it's essential to have conversations about sexual health with your partner(s), including discussions about HIV and STI status, before engaging in sexual activity. |  |
| 1. Does the IUD protect me to get HIV infection? | |
| No, an intrauterine device (IUD) does not protect against HIV infection or any other sexually transmitted infections (STIs). An IUD is a form of birth control that is highly effective at preventing pregnancy, but it does not provide any protection against HIV or STIs.  If you are sexually active and want to protect against HIV, it is important to consider the following methods:   - Using Condoms: When used consistently and correctly, both male and female condoms are effective at preventing HIV and certain other STIs. - Pre-Exposure Prophylaxis (PrEP): PrEP is a way for people who do not have HIV but who are at substantial risk of getting it to prevent HIV infection by taking a pill every day. - Getting Tested and Knowing Your Partner's HIV Status: Regular HIV testing ensures you know your status and can take appropriate measures to either protect yourself or manage your condition if you are HIV positive. - Reducing the Number of Sexual Partners: This can also help to reduce your risk of HIV infection.   As always, it's important to discuss your health with a healthcare provider to understand the best ways for you to protect against HIV and other STIs. |  |
| 1. May I protect myself from HIV infection with the pull-out method? | |
| The pull-out method, also known as withdrawal or "coitus interruptus," is not an effective method of preventing HIV transmission. This is because HIV can be present in pre-ejaculatory fluid (pre-cum), and there is always a risk that some semen may be released into the partner's body even before full ejaculation occurs.  To effectively reduce your risk of HIV transmission, you should consider the following methods:   - Using Condoms: When used correctly and consistently, condoms are one of the most effective ways to prevent HIV and other sexually transmitted infections (STIs). - Pre-Exposure Prophylaxis (PrEP): PrEP is a medication that, when taken daily by people who are at high risk for getting HIV, can prevent HIV infection. - Regular Testing: Regular testing for HIV and other STIs is an important part of sexual health, particularly for people who are sexually active with multiple partners. Knowing your status and that of your partner can help you make informed decisions about your sexual health. - Limiting Number of Sexual Partners and Avoiding High-Risk Behaviors: Reducing the number of sexual partners and avoiding practices like sharing needles can also significantly reduce your risk of HIV infection.   As always, it's important to have open, honest discussions with your healthcare provider about your sexual practices and concerns. They can provide you with personalized advice on the best ways to protect your health. |  |
| 1. Is it possible to get HIV infection using condoms? | |
| Condoms, when used consistently and correctly, are highly effective at preventing the sexual transmission of HIV. According to the U.S. Centers for Disease Control and Prevention, consistent use of condoms during sexual intercourse reduces the risk of HIV transmission by about 80%.  However, it's important to note that while condoms significantly reduce the risk, they do not entirely eliminate it. This is due to several factors:   1. **Incorrect Use:** Incorrect use of a condom can lead to its failure. This includes not using a condom throughout the entire sexual act, not leaving space at the tip, or not using sufficient lubrication. 2. **Breakage or Slippage:** Although it's rare, condoms can break or slip off during sex if not used properly. 3. **Quality and Expiry:** Using a damaged, expired, or poor-quality condom can increase the risk of breakage and hence HIV transmission. 4. **Non-Penetrative Acts:** Condoms protect against HIV during penetrative sex, but if you're engaging in sexual activities that do not involve penetration (such as oral sex or shared use of sex toys), condoms may not be used and can't provide protection.   To further reduce the risk of HIV transmission, consider additional strategies such as regular HIV testing, reducing the number of sexual partners, and using Pre-Exposure Prophylaxis (PrEP) if you're at high risk. |  |
| 1. May I prevent hiv by careful personal hygiene after sex? | |
| Cleaning or washing after sex can help maintain general hygiene, but it does not provide protection against HIV or other sexually transmitted infections (STIs). HIV is transmitted through specific body fluids—blood, semen, vaginal fluids, rectal fluids, and breast milk—and can enter the bloodstream through certain types of contact, such as vaginal or anal sex, or sharing needles. Once HIV is in your system, no amount of external cleaning can remove it.  Here are some effective ways to protect against HIV:   - Use Condoms: Condoms, when used consistently and correctly, provide one of the best forms of protection against HIV and other STIs during sexual activity. - Pre-Exposure Prophylaxis (PrEP): PrEP is a medication taken by people who are at high risk for getting HIV to prevent HIV infection. - Regular Testing: Knowing your own HIV status and that of your partner(s) is crucial. Regular testing allows for early detection, which can lead to early treatment and reduce the risk of transmitting HIV to others. - Reducing Number of Sexual Partners and Avoiding High-Risk Behaviors: Having fewer partners and avoiding practices such as sharing needles can significantly reduce your risk of HIV infection.   As always, consult with a healthcare provider for the most effective and personalized strategies for protecting your health. |  |
| 1. How can injecting drug users reduce their risk of contracting HIV? | |
| People who inject drugs (PWID) are at a high risk of contracting HIV due to the practice of sharing needles and other drug injection equipment. However, there are several strategies PWID can take to reduce their risk:   - Stop injecting drugs: This is the most effective way to eliminate the risk of HIV transmission through injection drug use. Drug counseling, treatment, and recovery services can help people stop or reduce their drug use. - Never share needles or equipment: If a person continues to inject drugs, it is crucial that they use a new, sterile needle each time and never share needles or equipment (such as cookers, cotton, or rinse water) with others. HIV can survive in a used needle for up to 42 days depending on temperature and other factors. - Syringe services programs (SSPs): These community-based programs provide access to sterile needles and syringes, facilitate safe disposal of used needles and syringes, and often offer other prevention materials and services. They also often provide referral to substance use disorder treatment programs.   Pre-exposure prophylaxis (PrEP): This is a strategy where HIV-negative individuals take antiretroviral medication daily to reduce their risk of contracting the virus. PrEP can be used by people who inject drugs, in addition to other prevention methods.   - Regular HIV Testing: Regular testing allows for early detection and treatment of HIV, which can improve health outcomes and prevent the spread of the virus to others. - Antiretroviral Therapy (ART): If a person who injects drugs is diagnosed with HIV, taking ART can suppress their viral load, improving their health and greatly reducing their chance of transmitting the virus to others.   It's important for individuals to consult with healthcare providers for personal advice and support. Additionally, community health initiatives and harm reduction programs often provide services tailored for people who inject drugs, including education, testing, and resources for safer drug use. |  |
| 1. How can a person who injects drugs or other substances obtain sterile injection equipment? | |
| There are several ways a person who injects drugs or other substances can obtain sterile injection equipment:   - Syringe Services Programs (SSPs): Also known as needle exchange programs, these community-based initiatives provide free sterile needles and syringes and often offer additional services such as safe disposal of used needles and syringes, testing for HIV and hepatitis C, education about safer injection practices, wound care, and referrals to substance use disorder treatment and social services. - Pharmacies: In many places, pharmacies sell sterile needles and syringes without a prescription. However, laws vary by location, and in some areas, pharmacies may refuse to sell needles or syringes without a prescription or proof of medical need. - Online: In some regions, sterile needles and syringes can be purchased online. However, individuals should be careful to purchase from a reputable supplier to ensure the equipment is indeed sterile. - Healthcare Providers: Some healthcare providers or health clinics may provide sterile needles and syringes to patients who inject drugs, particularly if they offer harm reduction services.   Remember, the most effective way to prevent HIV and other bloodborne infections among people who inject drugs is to stop injecting. However, if stopping isn't an option, using new, sterile equipment for each injection can greatly reduce the risk of infection. Also, never share needles or other injection equipment, even with trusted partners, as this is a high-risk behavior for HIV and other bloodborne diseases transmission. Always consult with a healthcare provider for advice tailored to specific circumstances and needs. |  |
| 1. Will cleaning IV-drug needles and syringes with bleach before using them prevent you from getting HIV? | |
| While cleaning needles and syringes with bleach can reduce the risk of HIV transmission, it does not eliminate it entirely. Therefore, it is not a foolproof method of preventing HIV infection. Bleach can kill the HIV virus, but the cleaning process must be done correctly and thoroughly, and even then, there can be room for error.  Furthermore, this method does not protect against other bloodborne pathogens like hepatitis C, which is more hardy than HIV and may not be killed by bleach.  The most effective way to prevent HIV and other bloodborne infections among people who inject drugs is to always use a new, sterile needle and syringe for each injection. This is often facilitated through needle exchange programs, which provide sterile needles and syringes to people who inject drugs.  In many places, these programs also provide other services such as education on safer injection practices, testing for HIV and hepatitis C, and referrals to substance use disorder treatment, including medication-assisted treatment.  If you or someone you know is injecting drugs, it's also important to consider seeking help for the underlying substance use disorder. There are effective treatments available, and healthcare providers can provide support and guidance. |  |
| 1. Why should you use condoms to prevent transmission when HIV is treatable? | |
| Even though HIV is treatable, there are several reasons why it's important to still use condoms and other prevention methods:   - Avoiding new infections: While antiretroviral therapy (ART) can manage HIV and reduce the risk of transmission, it's still possible for someone with the virus to transmit it to others, especially if they are not adhering to their treatment regimen or if they have not yet achieved an undetectable viral load. Condoms provide an additional layer of protection by preventing the exchange of bodily fluids during sex, which is the primary mode of HIV transmission. - Preventing other STIs: Condoms also protect against many other sexually transmitted infections (STIs), such as gonorrhea, chlamydia, and syphilis. These infections can be harmful on their own and can also increase the risk of acquiring or transmitting HIV. - Drug resistance: Some people with HIV can develop resistance to certain antiretroviral drugs over time, making those drugs less effective. The more people who have HIV, the more chance there is for drug-resistant strains to develop and spread. - Life-long treatment: HIV treatment is life-long and can have side effects. Some people experience challenges in adhering to the daily regimen of medication. - Access to treatment: Not everyone with HIV has access to the medicines they need due to factors like cost, healthcare access, and stigma. - Eliminating HIV: Using condoms and other prevention strategies are crucial in the global effort to eliminate new HIV infections.   So, while HIV is indeed treatable, it's best to avoid infection in the first place. Using condoms, getting regular testing, and considering other prevention options like pre-exposure prophylaxis (PrEP) if you're at high risk, are all strategies that can help. |  |
| 1. Should a woman douche after a condom breaks? | |
| No, douching (washing or cleaning out the vagina with water or other mixtures of fluids) after a condom breaks is not recommended as a method to prevent HIV or sexually transmitted infections (STIs). In fact, douching can actually increase the risk of contracting STIs, including HIV, for several reasons:   - Altering the Vaginal Flora: The vagina naturally maintains a balance of bacteria, known as the vaginal flora, which helps protect against certain infections. Douching can disrupt this balance, potentially leading to an overgrowth of harmful bacteria. - Pushing the Virus Further Up: If the virus is present, douching could potentially push it further up into the reproductive tract, increasing the risk of infection. - Causing Micro-Tears: Douching can lead to irritation and cause tiny tears in the vagina, potentially making it easier for the virus to enter the bloodstream.   If a condom breaks during sex and there's a risk of HIV exposure, post-exposure prophylaxis (PEP) is recommended. PEP is a prescription medicine that, if taken within 72 hours of exposure to HIV, can potentially prevent the virus from taking hold in your body. If a condom breaks, you should seek medical attention immediately to discuss the possibility of PEP.  Always, if you suspect you've been exposed to HIV or any other STI, it's crucial to get tested. Regular STI testing is a healthy practice for anyone who is sexually active, especially if condoms break or are not used consistently. |  |
| 1. What are the risks of getting HIV if you put on a condom after you've already started having sex? | |
| If a condom is only put on after sexual activity has already begun, there is a risk of HIV transmission, particularly if there has been an exchange of bodily fluids that can carry HIV, such as semen, vaginal fluids, or blood.  The primary reasons for using a condom are to prevent sexually transmitted infections (STIs) like HIV and to prevent unintended pregnancy. Condoms act as a barrier to prevent the exchange of fluids between partners. If a condom is not used from the start of sexual activity, there is a window of time during which transmission of HIV and other STIs can occur.  Here are some key points to remember about condom use:   - Use a new condom for every act of vaginal, anal, and oral sex—throughout the entire sex act (from start to finish). - Before any genital contact, put the condom on the tip of the erect penis with the rolled side out. - If the condom does not have a reservoir tip, pinch the tip enough to leave a half-inch space for semen to collect. Holding the tip, unroll the condom all the way to the base of the erect penis. - After ejaculation and before the penis gets soft, grip the rim of the condom and carefully withdraw. Then gently pull the condom off the penis, making sure that semen doesn't spill out. - Wrap the condom in a tissue and throw it in the trash where others won't handle it. - If you feel the condom break at any point during sexual activity, stop immediately, withdraw, remove the broken condom, and put on a new one.   Always ensure you're using condoms correctly and consistently for the best protection against HIV and other STIs. If you believe you've been exposed to HIV, seek medical attention immediately. Post-exposure prophylaxis (PEP) may be an option if taken within 72 hours of potential exposure. Regular HIV testing is also important for anyone who is sexually active. |  |
| 1. Is it safe for an HIV-positive person to have unprotected sex with another HIV-positive person? | |
| While it may seem that two HIV-positive individuals having sex wouldn't pose additional risk, this is not entirely accurate. Here are some reasons why unprotected sex between two HIV-positive individuals can still present risks:   - Superinfection: Superinfection occurs when a person with HIV gets infected with another strain of the virus. This can make the disease progress more rapidly and may also make the infection more difficult to treat if the new strain is resistant to the individual's current medications. - Different strains and drug resistance: Not all HIV viruses are the same. There are different strains and types of the virus, and some are resistant to certain antiretroviral drugs. Unprotected sex could potentially lead to one partner contracting a different, possibly drug-resistant strain of the virus from the other. - Other sexually transmitted infections (STIs): Unprotected sex increases the risk of transmitting other STIs. For someone who is already immunocompromised because of HIV, getting another STI can cause significant health problems. - Viral load: If one person has a higher viral load than the other, it could potentially increase the amount of virus in the body of the person with the lower viral load, which could potentially affect their health.   For these reasons, it's important for individuals with HIV to continue to practice safer sex, including using condoms correctly and consistently, even with other HIV-positive partners.  In addition, regular medical care and maintaining an undetectable viral load through effective antiretroviral therapy (ART) are crucial in preventing HIV transmission. A person with HIV who takes HIV medicine as prescribed and gets and stays virally suppressed or undetectable can stay healthy and has effectively no risk of sexually transmitting HIV to HIV-negative partners. |  |
| 1. What do I need to know about pregnancy and HIV infection? | |
| If you are pregnant or planning to become pregnant and are HIV-positive, there are certain things you should be aware of:   - Mother-to-Child Transmission: Without any intervention, HIV can be passed from a mother to her baby during pregnancy, childbirth, or breastfeeding. However, the risk of transmission can be greatly reduced with appropriate medical care and treatment. - Antiretroviral Therapy (ART): If you're HIV-positive and pregnant, you should be provided with antiretroviral therapy (ART) as soon as possible, if you're not already on it. ART can reduce your viral load, potentially to undetectable levels, significantly decreasing the risk of transmitting HIV to your baby. It's important to adhere strictly to the prescribed therapy. - Delivery Method: In some cases, your healthcare provider may recommend a cesarean section (C-section) to reduce the risk of mother-to-child transmission, particularly if your viral load is high. This is a decision made on a case-by-case basis. - Breastfeeding: HIV can be transmitted through breast milk. In countries where safe and affordable alternatives to breastfeeding are readily available, formula feeding is usually recommended to eliminate the risk. However, in countries where safe alternatives may not be available, the World Health Organization recommends exclusive breastfeeding alongside the mother's continued use of ART. - Regular Check-Ups: Regular check-ups and tests are vital during your pregnancy to monitor your health and the health of your baby. They also help to ensure that the HIV treatment is working effectively. - Neonatal ART: After birth, your baby may receive antiretroviral drugs for a short period to further reduce the risk of any potential HIV transmission. Your baby will also be tested for HIV multiple times in their first few months of life. - Remember, medical advice can vary depending on your individual circumstances, the stage of your pregnancy, your overall health, and the specific characteristics of your HIV infection. Always consult with a healthcare provider for personalized advice.   Above all, know that with appropriate treatment and care, you can have a healthy pregnancy and a healthy, HIV-negative baby. |  |
| 1. May I transmit HIV to my child during pregnancy? | |
| Yes, it is possible for a mother to transmit HIV to her baby during pregnancy, childbirth, or breastfeeding. This is called mother-to-child transmission (MTCT) or vertical transmission. However, with the right interventions, the risk of this transmission can be significantly reduced.  Here are some key interventions:   - Antiretroviral Therapy (ART): If you're HIV-positive and pregnant, you should start antiretroviral therapy (ART) as soon as possible if you aren't already on it. ART reduces the amount of virus in your body, often to undetectable levels, which significantly reduces the risk of transmitting HIV to your baby. - Safe Childbirth Practices: The risk of HIV transmission is also related to the type of delivery. If your viral load is high near your due date, your healthcare provider might recommend a scheduled cesarean section (C-section) to further decrease the risk. - Infant Antiretroviral Prophylaxis: After birth, your baby may receive antiretroviral drugs for a short period to reduce the risk of any HIV transmission that might have occurred during childbirth. - Avoidance of Breastfeeding: In situations where safe and acceptable alternatives to breast milk are available, mothers living with HIV should avoid breastfeeding to prevent postnatal transmission of HIV to their infants.   With these interventions, the risk of MTCT can be reduced to less than 2% in non-breastfeeding populations and to less than 5% in breastfeeding populations. Therefore, it's crucial to discuss your situation with a healthcare provider as soon as possible if you are pregnant or planning to become pregnant and are living with HIV. |  |
| 1. I have HIV; can I breastfeed my child? | |
| Breastfeeding can transmit HIV from a mother to her baby. However, the recommendations for breastfeeding when living with HIV vary depending on circumstances and location, particularly access to safe and sufficient infant formula.   - In resource-limited settings: The World Health Organization (WHO) recommends that if you're living with HIV and are on appropriate antiretroviral therapy (ART), you should exclusively breastfeed your infant for the first 6 months of life, then introduce appropriate complementary foods and continue breastfeeding up to the age of 12 months. ART can reduce the amount of the virus in your body to undetectable levels, thereby significantly reducing the risk of transmission. - In resource-rich settings: If you have access to safe and sufficient infant formula, it's generally recommended to formula feed from birth to completely eliminate the risk of mother-to-child transmission of HIV through breastfeeding.   It's important to have a conversation with your healthcare provider about your specific circumstances and what would be best for you and your baby. They can provide guidance based on the most recent guidelines, your personal health, and the resources available to you. |  |
| 1. Can two HIV-positive parents have an HIV-negative child? | |
| Yes, it is definitely possible for two HIV-positive parents to have an HIV-negative child. Advances in medical science have made this possible. The process generally involves the following:   - Antiretroviral Therapy (ART): Both parents should be on consistent antiretroviral therapy (ART) to reduce their HIV viral loads, ideally to undetectable levels. An undetectable viral load significantly reduces the risk of transmission. - Prevention of Mother-to-Child Transmission (PMTCT) During Pregnancy and Childbirth: The mother should be on ART throughout pregnancy and childbirth to maintain an undetectable viral load and to minimize the risk of transmitting HIV to the baby. - Cesarean Section (C-Section): If the mother's viral load is detectable near the time of delivery, a scheduled C-section may be recommended to further reduce the risk of HIV transmission during childbirth. - Avoidance of Breastfeeding or Safe Breastfeeding Practices: Depending on the specific circumstances and resources available, mothers living with HIV are often recommended to formula feed their babies to avoid the risk of transmission through breast milk. However, in settings where safe and adequate replacement feeding is not feasible, exclusive breastfeeding along with maternal ART is recommended for the first six months of life. - Infant Antiretroviral Prophylaxis: The newborn will typically be given antiretroviral medication for a period of time after birth as an additional safeguard. - Regular HIV Testing for the Baby: The baby will need to be tested for HIV at several points in time, typically at 2-3 weeks, 1-2 months, and again at 3-6 months, to ensure they remain HIV-negative.   With these measures in place, the risk of transmission can be reduced to less than 2%. However, it's important for both parents to work closely with their healthcare providers before and during pregnancy, as well as after the baby is born, to ensure the best outcomes for both mother and child. |  |
| 1. Can a couple in which one person is HIV positive conceive a baby without passing the virus to the uninfected partner? | |
| Yes, it's possible for a couple where one partner is HIV positive (often called serodiscordant or mixed-status couples) to conceive a baby without passing the virus to the HIV-negative partner. There are several strategies to reduce the risk of HIV transmission during conception:   - Treatment as Prevention (TasP): The HIV-positive partner takes antiretroviral therapy (ART) to maintain an undetectable viral load. Studies have shown that people with HIV who take ART as prescribed and maintain an undetectable viral load have effectively no risk of sexually transmitting HIV to their HIV-negative partners. This is known as Undetectable = Untransmittable, or U=U. - Pre-exposure Prophylaxis (PrEP): The HIV-negative partner can take a daily pill known as pre-exposure prophylaxis (PrEP) to reduce their risk of getting infected. When taken consistently, PrEP can reduce the risk of getting HIV from sex by about 99%. - Timed Conception: The couple can attempt to conceive during the HIV-negative partner’s ovulation period to reduce the number of sexual encounters and therefore the risk of exposure to HIV. - Sperm Washing: In some cases, couples might use assisted reproductive technologies. For instance, "sperm washing" is a procedure that separates sperm cells from seminal fluid, which is then used for insemination or in-vitro fertilization (IVF). - Assisted Reproductive Technology (ART): For couples where the male partner is HIV-positive and the female partner is HIV-negative, assisted reproductive techniques such as sperm washing followed by intrauterine insemination (IUI) or in vitro fertilization (IVF) can also be options.   Always consult with your healthcare provider or an HIV specialist to determine the best course of action for you and your partner. Each situation is unique, and what works best will depend on individual health, lifestyle factors, and personal preferences. |  |
| 1. What should I do if I am diagnosed with HIV infection during pregnancy? | |
| If you're diagnosed with HIV during pregnancy, the first thing to remember is not to panic. It's natural to be concerned, but with the right care and treatment, you can still have a healthy pregnancy and greatly reduce the risk of transmitting HIV to your baby. Here are some steps you should take:   - Seek Medical Care: If you haven't already, find a healthcare provider who is experienced in managing HIV and pregnancy. They will guide you through the process and develop a treatment plan that will be the most beneficial for both you and your baby. - Start Antiretroviral Therapy (ART): Starting treatment as soon as possible can help keep you healthy and significantly decrease the risk of transmitting HIV to your baby. Your healthcare provider will prescribe the most appropriate ART regimen for you. - Attend Regular Prenatal Visits: Regular check-ups allow your healthcare provider to monitor your health and your baby's health, the progress of your pregnancy, and the effectiveness of your HIV treatment. - Delivery Planning: Discuss with your healthcare provider about your delivery plan. In some cases, a cesarean section (C-section) may be recommended to further reduce the risk of transmission. - Avoid Breastfeeding: HIV can be transmitted through breast milk. If you live in a place where safe alternatives to breast milk are readily available, it is generally recommended to formula feed your baby to avoid the risk of transmission. - Infant Antiretroviral Prophylaxis: After birth, your baby will be given antiretroviral medication for a period of time as an additional safeguard. - Regular HIV Testing for the Baby: Your baby will be tested for HIV at several points to ensure they have not acquired the virus.   Remember, having HIV doesn't mean you can't have a healthy baby. With appropriate care and treatment, you can greatly reduce the risk of transmission and ensure the best outcomes for you and your baby. |  |
| 1. Can a woman with HIV have a vaginal delivery? | |
| Whether a woman with HIV can have a vaginal delivery depends on several factors, and this decision should be made with a healthcare provider who has experience in managing HIV during pregnancy. These factors include:   - Viral Load: The amount of HIV in the blood, known as the viral load, plays a key role in determining the mode of delivery. If a woman's viral load is low (typically defined as less than 1,000 copies of the virus per milliliter of blood), she may be able to have a vaginal delivery with a reduced risk of transmitting the virus to her baby. - Other Medical Conditions: Certain conditions, such as active genital herpes or a history of AIDS-related illnesses, might influence the mode of delivery. - Previous Birth History: Previous cesarean sections or other complications might necessitate a C-section in subsequent pregnancies. - Progress of Pregnancy and Labor: How labor progresses, and whether any complications develop, will also impact the decision. - Cesarean delivery (C-section) is often recommended for women with HIV if the viral load is high or unknown near the time of delivery, as it can help further reduce the risk of mother-to-child transmission of HIV.   However, it's important to note that with the right treatment, the risk of transmission can be less than 1%. Women with HIV should work closely with their healthcare provider to make the best decisions for their health and the health of their babies.  Please note, all of the above information is based on knowledge up to my training cut-off in September 2021, and more recent guidelines or research may provide different information. Always consult with your healthcare provider or an HIV specialist for the most current advice. |  |
| 1. Can the treatment for HIV damage the foetus? | |
| HIV treatment, also known as antiretroviral therapy (ART), is generally recommended for all people living with HIV, including pregnant women. ART reduces the amount of virus (viral load) in the body, which helps individuals living with HIV stay healthy and prevents transmission of HIV to others, including from a pregnant woman to her baby.  In terms of potential harm to the fetus, most antiretroviral drugs have been found to be safe for use in pregnancy. However, some specific drugs are known to have potential risks and are typically avoided during pregnancy. For example, as of my knowledge cutoff in September 2021, the antiretroviral drug efavirenz (Sustiva, also in the combination pill Atripla) is typically avoided during the first trimester of pregnancy due to potential risk of neural tube defects in the developing fetus.  The potential risks and benefits of any medication, including ART, should always be discussed with a healthcare provider. It's important that pregnant women living with HIV work closely with their healthcare providers to choose the best ART regimen for their individual situation.  In general, the benefits of treating HIV with ART far outweigh the potential risks, as untreated HIV can also pose serious risks to both the mother and baby, including the risk of transmitting HIV to the baby.  However, you should always consult with a healthcare provider for the most current and personal advice as my training only includes knowledge up to September 2021, and recommendations can change as new research becomes available. |  |
| 1. What is an HIV test? | |
| An HIV test is a medical test that determines if a person is infected with the Human Immunodeficiency Virus (HIV). There are several types of HIV tests, including:  Antibody Tests: These are the most common HIV tests. They work by looking for antibodies to HIV in a person's blood or oral fluid. Antibodies are proteins produced by the immune system in response to an infection. Rapid tests can provide results in as fast as 20 minutes or on the same day, while laboratory tests require blood samples and take longer to process.  Antigen/Antibody Tests: Also known as combination or combo tests, these detect both HIV antibodies and the HIV p24 antigen (a protein produced by the virus in the early stages of infection), which can help identify an HIV infection sooner than an antibody test alone. This test usually requires a blood sample from a vein.  Nucleic Acid Test (NAT): This test looks for the virus itself in the blood. It's very expensive and not typically used for screening individuals unless they recently had a high-risk exposure or a possible exposure and they have early symptoms of HIV infection.  HIV testing is an essential part of preventing the spread of the virus and treating it. If a person tests positive for HIV, it's important for them to start antiretroviral therapy (ART) as soon as possible. Regular HIV testing and knowing your status can help you make informed decisions about your health and the health of your partners. If you have any concerns or questions about HIV testing, you should speak to a healthcare provider. |  |
| 1. How long after possible exposure should I wait to be tested for HIV? | |
| The timing of an HIV test can be crucial because it can take some time for the immune system to produce enough antibodies for the HIV test to detect, which is a period known as the "window period."  For most people, an antibody test will detect HIV within a month of exposure, but in some cases, it may take up to three months. Antigen/Antibody tests, which are recommended by many experts, can detect the virus in most people within about 18-45 days post-exposure.  A nucleic acid test (NAT), which can detect the virus in the blood, can generally detect HIV in the blood 10 to 33 days after exposure, but it's not typically used for general screening due to its high cost.  While these are general guidelines, the specific timing can vary depending on the individual and the type of test. It's important to remember that if you test negative for HIV during the window period, you should be tested again after the window period ends to confirm your results.  If you believe you have been exposed to HIV, it is very important to seek medical advice immediately. If the exposure was recent, you might be a candidate for post-exposure prophylaxis (PEP), which may prevent an infection if started within 72 hours of exposure. You should also discuss ongoing HIV prevention strategies with your healthcare provider. |  |
| 1. Why should I get an HIV test? | |
| Getting tested for HIV is an important part of maintaining your overall health and the health of your partners. Here are several reasons why you should get an HIV test:  Knowledge is Power: Knowing your HIV status can give you peace of mind and help you make informed decisions about your health and your sexual activity.  Early Detection: If you are HIV-positive, early detection allows for early treatment. With today's advancements in antiretroviral therapy (ART), people with HIV can live long, healthy lives.  Preventing Transmission: If you know you are HIV-positive, you can take steps to protect your partners from becoming infected. This could include using condoms, taking your HIV medication regularly, which can reduce your viral load and the risk of transmission (a strategy known as "treatment as prevention"), or referring your partners for pre-exposure prophylaxis (PrEP).  Pregnancy and Childbirth: If you are pregnant or planning to become pregnant, knowing your HIV status can protect your health and the health of your baby. If a woman is HIV-positive and pregnant, there are treatments that can significantly reduce the risk of transmitting HIV to the baby.  It's a Part of Routine Healthcare: The Centers for Disease Control and Prevention (CDC) recommends that everyone between the ages of 13 and 64 get tested for HIV at least once as part of routine healthcare and more frequently if you're at higher risk.  Remember, while HIV testing is voluntary and you have the right to decline it, getting tested is a key part of preventing the spread of the virus and improving your health outcomes if you are HIV-positive. If you have any concerns or questions about HIV testing, you should speak to a healthcare provider. |  |
| 1. Where can I get tested? | |
| There are several places where you can get tested for HIV, and the options can depend on your location and specific needs. Some of these places include:   - Primary Care Doctor's Office: Your regular doctor can order an HIV test and may be able to provide counseling and support if the test comes back positive. - Sexual Health Clinics: These specialized clinics offer confidential and often free or low-cost testing services. - Community Health Centers: Many community health centers offer HIV testing. These are often present in urban and some rural areas. - Planned Parenthood Health Centers: Planned Parenthood provides a range of sexual health services, including HIV testing. - Hospitals and Emergency Rooms: Many hospitals offer HIV testing, particularly if you are there for a related health concern. - Mobile Testing Units: Some organizations have mobile testing units that travel to communities to provide free, rapid HIV tests. - Pharmacies: Some pharmacies offer free HIV testing at certain times of the year, and many sell home test kits you can use privately. - Home Testing Kits: Over-the-counter HIV home testing kits are available for purchase online and in drugstores. They provide rapid results within 20-40 minutes. - HIV Service Organizations: Some organizations dedicated to HIV/AIDS also offer testing services. - Substance Abuse Treatment Programs: For individuals with substance use issues, testing may be available at treatment centers. - Public Health Departments/Local Health Departments: These government agencies often provide testing services, particularly to people who might not otherwise have access to healthcare.   Keep in mind that you should always seek testing from a reputable source to ensure that the test is accurate and that your information will be kept confidential. In the United States, you can visit www.hiv.gov/locator to find a testing site near you. If you are not in the U.S., check the websites of your local health department or HIV/AIDS organizations for similar resources. |  |
| 1. What does it mean if I test negative for HIV? | |
| Testing negative for HIV means that at the time of testing, there were no detectable antibodies or antigens to HIV in your body, and you are likely not infected with the virus.  However, it's important to understand that there is a "window period" in HIV testing. This is the period of time between when a person may have been exposed to HIV and when a test can accurately detect the infection. Most modern HIV tests can detect the virus as early as 2 weeks after exposure, but in some cases, it may take up to 3 months for the body to produce enough antibodies for the test to detect.  If you tested negative during this window period, you might get a false-negative result, which means the test says you don't have HIV when you actually do. That's why it's recommended to get retested three months after a potential exposure if your test result is negative, particularly if you've engaged in behavior that puts you at risk for contracting HIV.  Also, it's crucial to remember that testing negative for HIV does not mean you're immune to the virus. You can still become infected if you engage in risky behaviors, such as having unprotected sex or sharing needles. Ongoing preventive measures like using condoms correctly and consistently, reducing the number of sexual partners, never sharing needles, and considering pre-exposure prophylaxis (PrEP) if you're at high risk, are key in protecting against HIV.  Always consult with a healthcare provider to discuss your testing and prevention options, and to interpret your test results accurately. |  |
| 1. What does it mean if I test positive for HIV? | |
| Testing positive for HIV means that the test has detected the presence of HIV antibodies or antigens in your body, suggesting that you have been infected with the virus.  It's important to know that if you get a positive result from a rapid, at-home, or initial laboratory test, you will need to have a follow-up confirmatory test to make sure the first test was accurate. This is because, while HIV tests are highly accurate, no test is 100% perfect, and there can occasionally be false-positive results.  If the confirmatory test also comes back positive, it means that you have HIV. While this news can be upsetting, know that HIV is a manageable condition and many people with HIV live long, healthy lives. There have been significant advancements in treatment, and most people with HIV who are on regular antiretroviral therapy can achieve an undetectable viral load -- meaning the virus is not detectable in their blood.  Achieving an undetectable viral load not only helps you stay healthy, but it also means you can't sexually transmit the virus to an HIV-negative partner, a concept known as "U=U" or "Undetectable equals Untransmittable".  If you test positive, you should be linked to an HIV care provider who can discuss treatment options with you. Starting antiretroviral therapy as soon as possible is recommended for all people with HIV.  In addition, emotional and mental health support can be beneficial, whether through professional counseling, peer support groups, friends and family, or other means. Living with HIV does come with challenges, but with the right care and support, most can be effectively managed. |  |
| 1. How are commons false negatives and false positives tests for HIV? | |
| False negatives and false positives in HIV testing, while uncommon, can occur. These are the terms used when the test results do not accurately reflect whether a person has HIV.   - False Negative: This means the test result is negative, but the person actually has HIV. False negatives typically occur during the "window period," which is the time between when a person is exposed to HIV and when the body produces enough antibodies or antigens for the test to detect. Depending on the type of test used, this window period can be anywhere from 10 days to three months. If someone gets tested during this window period, they may receive a false negative result even though they are infected. - False Positive: This means the test result is positive, but the person does not have HIV. False positives are less common but can happen. They can be caused by factors such as the presence of other antibodies in the blood that react with the test, certain medical conditions, or technical issues with the testing process.   It's important to note that when an initial HIV test gives a positive result, a follow-up confirmatory test is always done. These follow-up tests are highly accurate and help ensure that people are not falsely diagnosed with HIV.  Overall, HIV tests are extremely accurate. The most commonly used tests have a sensitivity of about 99.5%, which means they can correctly identify about 99.5% of people who have HIV. The specificity of these tests is also about 99.5%, meaning they can correctly identify about 99.5% of people who do not have HIV.  Getting tested regularly and understanding the limitations and accuracy of HIV tests is crucial, especially for those at higher risk of HIV infection. Always consult a healthcare provider for advice tailored to your specific circumstances and needs. |  |
| 1. What do "inconclusive" test results mean on a home HIV test? | |
| "Inconclusive" test results mean that the test was not able to definitively determine whether you have HIV or not. An inconclusive result is not common, but it can occur for several reasons:  Testing during the window period: The window period refers to the time between potential exposure to HIV and when the test can accurately detect it. If you get tested too soon after a possible exposure, the test might not pick up the infection.  Technical errors: There might be an issue with the test itself, such as a manufacturing defect, or an error may have occurred while performing the test.  Non-specific reactions: Sometimes, substances in your blood can cause the test to react even if HIV is not present.  If you get an inconclusive result on a home HIV test, you should take the following steps:  Retest: Follow up with another test. You can either take another home test or visit a healthcare provider for a test. It's generally a good idea to wait a few weeks before retesting, especially if you might still be within the window period.  Consult a healthcare provider: Discuss your results and your potential risk of HIV with a healthcare provider. They can help you understand your results and decide on the next best steps.  Remember, it's crucial to know your HIV status. If you're sexually active or have other risk factors for HIV, regular testing should be a part of your healthcare routine.  If you're at high risk for HIV, consider talking to a healthcare provider about preventive strategies, such as pre-exposure prophylaxis (PrEP), a medication that can significantly reduce your risk of getting HIV. |  |
| 1. How Accurate Are In-Home HIV Tests? | |
| In-home HIV tests, also known as home HIV test kits, can provide a convenient way to test for HIV at home. There are a couple of FDA-approved home HIV tests available in the United States as of my knowledge cutoff in 2021. These include the OraQuick In-Home HIV Test and the Home Access HIV-1 Test System.   - OraQuick In-Home HIV Test: This is a rapid test that involves swabbing your mouth for an oral fluid sample. You can read the results within 20 to 40 minutes. According to the manufacturer, the OraQuick test has a sensitivity of about 91.7%, meaning it can identify about 91.7% of people who have HIV. Its specificity is about 99.9%, meaning it can correctly identify about 99.9% of people who do not have HIV. - Home Access HIV-1 Test System: This test involves pricking your finger to get a blood sample that you send to a laboratory. You then call for results as early as the next business day. The Home Access test has a sensitivity of about 99.9% and a specificity of about 99.5%.   While these home tests can be very accurate, they are not perfect. For example, like all HIV tests, they have a window period. If you take the test too soon after a potential exposure, you might get a false-negative result. The window period for the OraQuick test is about three months, while for the Home Access test, it's about six weeks.  False positives can also occur, though they are less common. If you get a positive result with a home test, it's crucial to have a follow-up confirmatory test at a clinic or doctor's office to make sure the result is accurate.  As always, consult with a healthcare provider for advice tailored to your specific circumstances and needs. And remember, HIV testing is just one part of a comprehensive approach to HIV prevention, which also includes strategies like safer sex practices and potentially using pre-exposure prophylaxis (PrEP) if you're at high risk. |  |
| 1. How accurate are rapid oral HIV tests? | |
| Salivary HIV tests, also known as oral fluid HIV tests or rapid oral HIV tests, are a convenient, less invasive type of test for HIV. The most common brand of this type of test is the OraQuick In-Home HIV Test, which is available over-the-counter.  The OraQuick test works by detecting antibodies — proteins that the body produces in response to infections — to HIV in oral fluid. The test involves swabbing the gums and placing the swab in a tube where it will be able to react with a solution to detect HIV antibodies. Results are typically available in 20 to 40 minutes.  According to the Centers for Disease Control and Prevention (CDC) and the manufacturer's information, the OraQuick In-Home HIV Test has a sensitivity of approximately 91.7%. This means that the test correctly identifies HIV in about 91.7% of people who are infected. This also means that the test could give a false negative result to as many as 1 in 12 people who are actually infected with HIV.  The OraQuick test has a specificity of approximately 99.9%, meaning it correctly identifies about 99.9% of people who are not infected with HIV. In other words, about 1 in 1,000 people might get a false positive result.  It's important to note that like all HIV tests, the oral fluid test has a window period. This is the time between when a person may have been exposed to HIV and when a test can accurately detect the virus. If you take the test during this window period, it may not detect HIV even if you are infected. The window period for the OraQuick In-Home HIV Test is around three months.  Remember that any positive result from a rapid HIV test (including salivary tests) must be confirmed with a follow-up blood test in a clinical setting. |  |
| 1. How often should I get tested? | |
| The frequency of HIV testing depends on your individual risk factors, lifestyle, and sexual behavior. The Centers for Disease Control and Prevention (CDC) recommends that:   - Everyone between the ages of 13 and 64 get tested for HIV at least once as part of routine health care. - Those at high risk get tested more frequently. The CDC recommends annual testing if you are sexually active and have had more than one partner since your last HIV test, or if you have other risk factors. - Sexually active gay and bisexual men may benefit from more frequent testing, such as every 3 to 6 months.   People who are at higher risk include:   - Men who have sex with men. - People who have had sex with an HIV-positive partner. - People who have had multiple sex partners since their last HIV test. - People who inject drugs or share needles, syringes, rinse water, or other drug preparation equipment. - People who exchange sex for drugs or money. - People diagnosed with, or seeking treatment for, another sexually transmitted disease. - People who have been diagnosed with or treated for hepatitis or tuberculosis (TB). - People whose sexual partners have participated in any of these activities.   It's important to consult with a healthcare provider for advice tailored to your specific circumstances and needs. Additionally, using barrier methods such as condoms, practicing safer sex, and in some cases, using medications such as pre-exposure prophylaxis (PrEP) can help reduce the risk of HIV infection. |  |
| 1. How soon after risky sex can you be 100% sure you are clear of HIV? | |
| There isn't a set period of time after which you can be 100% certain that you're clear of HIV following a potential exposure. However, most healthcare providers recommend getting tested at specific intervals to be reasonably certain that you are not infected.  Here's a general timeline:   - Within 72 hours of exposure: If you think you've been exposed to HIV within the past 72 hours, you might be a candidate for post-exposure prophylaxis (PEP), a course of drugs that can potentially prevent HIV infection if started soon after exposure. You should contact a healthcare provider immediately if this is the case. - 4 weeks to 3 months after exposure: Modern tests called combined antigen/antibody tests can detect HIV as early as 2 weeks to a month after infection, but it can take up to three months for them to be completely accurate. - 3 months after exposure: The window period for many HIV tests is three months, and the CDC recommends retesting at this time for conclusive results. By three months, about 97% of people will have developed enough antibodies to test positive for HIV if they are infected.   Remember, though, that no test can detect HIV immediately after infection. There's always a "window period" between the time of infection and when a test can detect the virus.  If you think you may have been exposed to HIV, you should contact a healthcare provider right away. They can provide you with the most appropriate advice for your situation. Regardless of your test results, it's important to consistently use safer sex strategies to protect yourself and others from HIV and other sexually transmitted infections. |  |
| 1. What is PrEP? | |
| Pre-exposure prophylaxis, or PrEP, is a preventive strategy used to reduce the risk of HIV infection in people who are at high risk. This strategy involves the regular use of antiretroviral medications.  As of my last training data in September 2021, the two medications approved for use as PrEP are:   - Truvada: A combination of two medicines, tenofovir and emtricitabine. - Descovy: A combination of two medicines, emtricitabine and tenofovir alafenamide. As of my training data, Descovy is not approved for use as PrEP in individuals at risk of HIV from receptive vaginal sex.   When taken consistently and correctly, PrEP can significantly reduce the risk of acquiring HIV. PrEP is even more effective when it is combined with other prevention methods, such as condom use.  PrEP does not protect against other sexually transmitted infections (STIs) and does not prevent pregnancy. Therefore, it is not a substitute for condoms or other forms of birth control.  Before starting PrEP, you should confirm that you are HIV-negative, as taking PrEP when already infected with HIV can lead to drug resistance. Regular follow-up appointments (typically every 3 months) are needed while taking PrEP, to monitor for any side effects, check kidney function, and ensure the continued effectiveness of the medication.  It's always recommended to consult with your healthcare provider to discuss the risks and benefits and determine whether PrEP is a good option for you. |  |
| 1. What is the difference between PrEP and PEP? | |
| PrEP (pre-exposure prophylaxis) and PEP (post-exposure prophylaxis) are both strategies to prevent HIV infection using antiretroviral medications, but they are used in different situations.   - PrEP is a daily medication taken by people who are at high risk for HIV to prevent getting the virus. It's used by people who are HIV-negative and want to remain that way. PrEP should be taken consistently every day to ensure maximum effectiveness. When taken properly, PrEP can reduce the risk of getting HIV from sex by about 99%. - PEP, on the other hand, is a medication taken after potential exposure to HIV. It's a series of pills you start taking as soon as possible after you may have been exposed to HIV, and you keep taking it once or twice daily for 28 days. PEP should be used only in emergency situations and must be started within 72 hours after a recent possible exposure to HIV.   While both PrEP and PEP can be effective in preventing HIV, they do not protect against other sexually transmitted infections (STIs) or pregnancy. Other prevention methods, like condoms, should be used in combination with PrEP or PEP to protect against STIs and/or pregnancy. It's also important to note that while PrEP and PEP can greatly reduce the risk, they are not 100% effective in preventing HIV infection. |  |
| 1. What it the efficacy of PrEP? | |
| Pre-exposure prophylaxis, or PrEP, is a very effective method of preventing HIV infection if used correctly. According to the Centers for Disease Control and Prevention (CDC), when taken daily as directed, PrEP can reduce the risk of getting HIV from sexual activity by about 99%.  It's important to note that the effectiveness of PrEP can be reduced if it's not taken consistently. Missing doses can significantly decrease its effectiveness. PrEP also does not provide protection against other sexually transmitted infections (STIs), so condoms should still be used for comprehensive protection.  PrEP also works as part of a comprehensive strategy to manage and prevent the spread of HIV, which can include routine testing, condom use, and treatment for those already infected with the virus.  As always, individuals should consult their healthcare provider to discuss the risks and benefits and determine whether PrEP is a good option for them. It's also important for individuals taking PrEP to have regular check-ups with their healthcare provider, as PrEP can have side effects and may impact kidney function. |  |
| 1. Is it safe assuming PrEP? | |
| Pre-exposure prophylaxis (PrEP) is a medication taken by people who are at a high risk of getting HIV, in order to prevent HIV infection. As of my knowledge cutoff in September 2021, two medications are approved for PrEP: Truvada and Descovy.  In general, PrEP is considered safe and well-tolerated. It's highly effective at preventing HIV transmission when taken consistently. According to the U.S. Centers for Disease Control and Prevention, PrEP reduces the risk of getting HIV from sex by about 99% when taken daily.  However, like all medications, PrEP can have side effects. The most common side effects are mild, like nausea, but usually go away over time. In some cases, PrEP can cause more serious side effects, such as kidney problems, bone density loss, or liver problems.  Before starting PrEP, doctors typically perform tests to ensure that a person is not currently HIV-positive, as PrEP medicines are not enough to treat HIV. They also monitor kidney function and other health parameters during PrEP use.  It's also important to remember that while PrEP is highly effective at preventing HIV, it does not protect against other sexually transmitted infections (STIs) or pregnancy. It should be used in conjunction with other prevention strategies like condoms for more comprehensive protection.  If you are considering PrEP, discuss it with your healthcare provider. They can provide guidance based on your personal medical history and lifestyle. They can also help you understand how to use PrEP effectively. |  |
| 1. What is dapivirine ring? | |
| The dapivirine vaginal ring is a female-initiated method of HIV prevention. It's a flexible, silicone ring that is inserted into the vagina once a month, where it slowly releases the antiretroviral drug dapivirine over time.  The dapivirine ring was developed by the nonprofit International Partnership for Microbicides (IPM) as a discreet and long-acting method to reduce women's risk of HIV infection. The ring provides a sustained and controlled release of the drug to the site of potential infection, with minimal systemic absorption.  Clinical trials of the dapivirine ring have shown that it can reduce the risk of HIV infection in women. As of my knowledge cutoff in September 2021, the ring has been approved by the European Medicines Agency and is under review by other regulatory authorities, including the U.S. Food and Drug Administration.  However, it's important to note that like other HIV prevention methods, the dapivirine ring is not 100% effective and does not protect against other sexually transmitted infections or pregnancy. Therefore, it should be used as part of a comprehensive HIV prevention strategy. |  |
| 1. What is the efficacy of dapivirine ring? |  |
| The efficacy of the dapivirine vaginal ring for HIV prevention has been evaluated in multiple large-scale clinical trials. In two Phase III trials, The Ring Study and ASPIRE, the dapivirine ring reduced the risk of HIV infection by approximately 30% overall when compared to a placebo.  However, the studies also found that the dapivirine ring's effectiveness varied among different groups of women. For example, women who were over 21 and used the ring more consistently appeared to have higher levels of protection (over 50% in some analyses). The effectiveness was lower among younger women who appeared to use the ring less consistently.  Additionally, a follow-up study called HOPE/MTN-025 and DREAM showed that with higher adherence and uptake, the dapivirine ring may reduce HIV risk by 50% or more.  These findings underscore the importance of consistent use and adherence to the prescribed regimen for achieving the maximum protective benefit from the dapivirine ring. As with other forms of HIV prevention, the dapivirine ring should be part of a comprehensive HIV prevention strategy that includes other measures such as condom use, regular HIV testing, and treatment for partners with HIV. It's important to note that the dapivirine ring does not protect against other sexually transmitted infections or prevent pregnancy.  As of my knowledge cutoff in September 2021, more research is underway to continue to assess the long-term safety and efficacy of the dapivirine ring. It's always recommended to consult with a healthcare provider to discuss HIV prevention strategies and options. |  |
| 1. Which tests should I do before starting PrEP? | |
| Before starting pre-exposure prophylaxis (PrEP), a regimen designed to reduce the risk of contracting HIV, there are several tests that healthcare providers typically recommend:   - HIV Test: An HIV test is essential before starting PrEP. PrEP is for individuals who are at high risk for contracting HIV but who are currently HIV-negative. Starting PrEP when already HIV positive can lead to drug resistance. - Kidney Function Tests: Because the medications used in PrEP can affect kidney function, tests to measure how well your kidneys are working are necessary. - Hepatitis B and C Tests: These tests are important because the medications used in PrEP can also affect hepatitis B and C infections. - STI Screening: Since individuals at high risk for HIV are also often at risk for other sexually transmitted infections (STIs), a comprehensive STI screening is typically recommended. - Pregnancy Test: If relevant, a pregnancy test might be done. PrEP drugs are generally considered safe in pregnancy, but the decision to use PrEP during pregnancy should be made in consultation with a healthcare provider.   It's important to remember that regular follow-up appointments will be needed while on PrEP. These visits typically include repeat HIV testing, STI screening, and monitoring of kidney function.  As always, consult your healthcare provider for advice tailored to your specific situation. Please note that my training only includes information available as of September 2021, and there may have been advancements or changes in the understanding of this subject since that time. |  |
| 1. How often do I have to check when on PrEP? | |
| When you're taking pre-exposure prophylaxis (PrEP) for HIV prevention, regular check-ups with your healthcare provider are essential. Here's a general outline of what you might expect, based on guidelines available as of September 2021:   - HIV Testing: You should get tested for HIV every 3 months. PrEP is only for people who are HIV-negative, and taking it while HIV-positive can lead to drug-resistant strains of HIV. Therefore, it's important to confirm that you remain HIV-negative while on PrEP. - Kidney Function Tests: Your healthcare provider should monitor your kidney function before you start PrEP and then at least every six months while you're taking it. - STI Screening: Sexually transmitted infections (STIs) should be checked for every 3-6 months, depending on your risk factors and symptoms. - Prescription Refills and Adherence Counseling: PrEP needs to be taken consistently to work effectively, so regular visits (often every 3 months) are needed for prescription refills and to discuss any issues with taking the medication regularly. - Pregnancy Testing: If applicable, regular pregnancy tests may be needed, based on your reproductive plans and contraceptive use.   Please remember that these are general guidelines and your healthcare provider might have a different plan based on your specific situation. Also, keep in mind that my training only includes information available up until September 2021, and there may have been advancements or changes in the understanding of this subject since that time. Always consult with a healthcare provider for the most current and personal advice. |  |
| 1. Do I have to take PrEP every day? | |
| Yes, for the most part, the recommended use of pre-exposure prophylaxis (PrEP) for HIV prevention involves taking the medication daily. The efficacy of PrEP is significantly higher when it is used consistently as prescribed.  There is also an approach known as "on-demand" or "event-based" PrEP, where the medication is taken before and after sexual activity instead of every day. This approach, also known as 2-1-1 dosing, involves taking two pills of PrEP 2-24 hours before anticipated sexual activity, then one pill 24 hours after the first dose, and one more pill 48 hours after the first dose. This strategy is primarily recommended for men who have sex with men. It's important to note that this approach requires a good understanding of the regimen and the ability to anticipate sexual activity.  Regardless of the regimen, PrEP should always be used under the guidance of a healthcare provider, who can provide advice tailored to an individual's lifestyle and risk factors. And remember, PrEP is not effective against other sexually transmitted infections, so it should be used in conjunction with other protective measures, such as condom use. |  |
| 1. If I do PrEP, am I also protected against other infections? | |
| No, pre-exposure prophylaxis (PrEP) does not provide protection against other sexually transmitted infections (STIs) such as gonorrhea, chlamydia, or syphilis. PrEP is specifically designed to prevent HIV infection.  If you're sexually active and at risk for STIs, regular testing and the use of barrier methods, such as condoms, can help reduce your risk. Vaccinations are also available for certain STIs like human papillomavirus (HPV) and Hepatitis A and B.  It's important to have regular check-ups with your healthcare provider and discuss your sexual health openly, even if you're taking PrEP. They can provide advice tailored to your specific needs and circumstances, and help ensure you're protecting yourself as effectively as possible. |  |
| 1. I am assuming PrEP. Do I still need to use condoms? | |
| Yes, it is still recommended to use condoms even if you're taking pre-exposure prophylaxis (PrEP). This is for a few reasons:   - Protection against other sexually transmitted infections (STIs): While PrEP is very effective at preventing HIV, it does not protect against other STIs like chlamydia, gonorrhea, syphilis, herpes, or HPV. Condoms can significantly reduce the risk of most of these infections. - Additional protection against HIV: Although PrEP greatly reduces the risk of HIV infection, no method is 100% effective. Using condoms consistently and correctly provides an additional layer of protection. - Prevention of unplanned pregnancies: If you're having heterosexual sex and there's a possibility of pregnancy, condoms can provide effective contraception. PrEP does not have any contraceptive effect.   Therefore, while PrEP is a powerful tool in the fight against HIV, it should be used as part of a comprehensive approach to sexual health and safety, including regular STI testing and condom use. |  |
| 1. What might happen if I miss doses? | |
| PrEP, or pre-exposure prophylaxis, is most effective when it's taken consistently as prescribed by your healthcare provider. Missing doses can significantly reduce PrEP's effectiveness in preventing HIV.  If you miss a dose of PrEP, take it as soon as you remember unless it's almost time for your next dose. Don't double up on your next dose if you've missed one. Try to get back into your routine as soon as possible.  If you're finding it difficult to remember to take your PrEP medication regularly, it's worth speaking with your healthcare provider. They may be able to provide you with strategies to help you remember, such as setting reminders on your phone, or pairing your medication with a daily activity like eating a meal.  Remember, PrEP is just one part of a comprehensive strategy to prevent HIV. Other strategies include regular testing, safer sex practices like using condoms, and getting regular check-ups for other sexually transmitted infections. If you've had a potential recent exposure to HIV, particularly if you've missed several doses of PrEP, speak to your healthcare provider right away as you might benefit from post-exposure prophylaxis (PEP). |  |
| 1. What should I do if I miss a dose of PrEP? | |
| If you miss a dose of pre-exposure prophylaxis (PrEP), it's recommended that you take the missed dose as soon as you remember, unless it's almost time for your next dose. In that case, skip the missed dose and take the next one at the regular time. Do not double up on your next dose to make up for the one you missed.  If you're having difficulty remembering to take your medication, consider setting reminders on your phone or linking your medication routine with a daily habit, like brushing your teeth.  Consistency is key with PrEP, as its effectiveness at preventing HIV decreases if not taken regularly. If you frequently miss doses or struggle with daily adherence, discuss this with your healthcare provider. They may have additional strategies for improving adherence, or could consider whether a different HIV prevention strategy might be a better fit for you.  If you have had a potential HIV exposure, particularly if you've missed several doses of PrEP, contact a healthcare provider immediately. You may need post-exposure prophylaxis (PEP), which can prevent HIV infection if started within 72 hours of exposure. |  |
| 1. What are the side effects of PrEP? | |
| Pre-exposure prophylaxis (PrEP) is generally well tolerated, but like any medication, it can have side effects. Some people taking PrEP may experience:   - Gastrointestinal symptoms: These include nausea, vomiting, abdominal pain, and diarrhea. These symptoms often occur when a person first starts taking PrEP, but they usually improve over time as the body adjusts to the medication. - Weight loss: Some people may experience minor weight loss when they start PrEP, especially if they're experiencing gastrointestinal side effects. - Headaches: Some people report mild headaches when they first start PrEP. - Fatigue: Some people report feeling tired or low on energy when they first start PrEP. - Changes in kidney function: PrEP can affect kidney function in some people, so regular monitoring of kidney function is important. - Decrease in bone mineral density: Some studies have shown a slight decrease in bone mineral density with PrEP use, but this does not appear to result in a higher risk of fractures.   These side effects are not experienced by everyone and can often be managed with the help of a healthcare provider. Most people taking PrEP have no side effects or only mild side effects that go away over time.  If you're taking PrEP and you're experiencing side effects that are severe, don't go away, or are affecting your quality of life, it's important to discuss these with your healthcare provider. They can help determine whether these side effects are related to PrEP and what can be done to manage them.  Keep in mind that the benefits of PrEP in preventing HIV usually outweigh these potential side effects, especially for people at high risk of HIV infection. If you have concerns about side effects, discuss them with your healthcare provider. |  |
| 1. Can I take PrEP with my other medicines? | |
| PrEP, or pre-exposure prophylaxis, is generally safe to take with most other medications. However, like with any medication, there can be interactions with certain other drugs that could affect the efficacy of either medication or increase the likelihood of side effects.  Common medications that might interact with PrEP include certain antifungal or antibiotic medications, some antiviral medications used for hepatitis B and C, and certain antacid medications. PrEP may also interact with some recreational drugs, including ecstasy and amphetamines.  It's important that you inform your healthcare provider about all the medications, supplements, and recreational drugs you are taking so they can evaluate for any potential drug interactions. Your provider will also regularly monitor your kidney function while you're taking PrEP, as certain other medications can increase the risk of kidney problems.  It's also worth noting that if you are taking hormonal birth control, PrEP does not interfere with the effectiveness of these contraceptives. Similarly, hormonal contraceptives do not affect the efficacy of PrEP.  In summary, while it's likely safe to take PrEP with your other medications, you should always discuss your full medication list with your healthcare provider to ensure there are no harmful interactions. |  |
| 1. Does PrEP interfere with hormone therapy for transgender patients? | |
| As of my knowledge cutoff in September 2021, current research suggests that PrEP (pre-exposure prophylaxis) and hormone therapy can be taken together without affecting the levels or effectiveness of either medication.  Studies have shown that feminizing hormone therapy (estrogen and anti-androgens) does not impact the levels of tenofovir or emtricitabine (the two medications in most PrEP regimens) in the body. Similarly, these medications do not appear to affect hormone levels.  However, individual responses can vary, and all medications have the potential for side effects or interactions. Therefore, it's important for anyone starting a new medication, including PrEP, to discuss this with their healthcare provider, especially if they're also taking hormone therapy.  The provider will monitor for potential drug interactions and side effects, and can adjust the treatment plan if necessary. Regular follow-up appointments are important to monitor kidney function and HIV status, among other things.  While PrEP is highly effective in preventing HIV, it does not protect against other sexually transmitted infections. Therefore, using condoms and getting regularly tested for STIs is still important.  Please consult with your healthcare provider or a pharmacist for the most current information as this field is continuously evolving. |  |
| 1. Does PrEP interfere with birth control pills? | |
| No, as of the current knowledge cut-off in September 2021, pre-exposure prophylaxis (PrEP) does not interfere with hormonal birth control pills. The components of PrEP, tenofovir and emtricitabine, do not affect the levels or effectiveness of hormonal contraceptives. Similarly, hormonal contraceptives do not affect the effectiveness of PrEP in preventing HIV infection.  However, it's always important to let your healthcare provider know about all the medications you're taking, including birth control pills, so they can monitor for any potential drug interactions.  Also, keep in mind that while PrEP is effective at preventing HIV, it doesn't protect against other sexually transmitted infections or prevent pregnancy. Therefore, using condoms and/or other methods of contraception (like birth control pills) is still important if those are concerns for you.  Remember that health guidelines can change as new research becomes available, so it's always a good idea to have these conversations with your healthcare provider or a trusted medical professional. |  |
| 1. Will I have to take PrEP for the rest of my life? | |
| Not necessarily. Pre-exposure prophylaxis (PrEP) is a preventive strategy for people who are currently at very high risk of getting HIV. Whether you take PrEP long term will depend on several factors:   - Risk Level: You may only need to be on PrEP during periods of your life when you are at high risk of getting HIV. For example, if you have a partner with HIV, are having sex without condoms with partners of unknown HIV status, or are using injectable drugs. - Lifestyle Changes: If your risk level changes because of changes in your behaviors (for example, if you start using condoms consistently, or if your partner with HIV achieves and maintains an undetectable viral load), you might decide to stop taking PrEP. - Other Health Factors: For example, if you experience negative side effects from the medication, you and your healthcare provider might decide that it is best for you to stop taking PrEP.   It's crucial to have regular check-ins with your healthcare provider to discuss your risk level and whether you should continue on PrEP. This should be a shared decision-making process between you and your healthcare provider, considering your personal circumstances, risk level, and lifestyle.  Also remember, it is important to continue taking PrEP for as long as you are at risk and not to stop taking it without discussing with your healthcare provider, as stopping PrEP can leave you unprotected from HIV. |  |
| 1. What if I want to stop PrEP? | |
| If you want to stop taking PrEP (pre-exposure prophylaxis), it's important to discuss this decision with your healthcare provider. They can guide you through the process and ensure that you understand the implications. Here are some key things to consider:  Risk Assessment: Your healthcare provider can help you reassess your current risk of acquiring HIV. If your risk has decreased due to changes in your behavior, it may be appropriate to stop taking PrEP.  Timing: If you're considering stopping PrEP, the timing can be important. For example, PrEP should be taken for a certain period of time after the last potential exposure to HIV. Your healthcare provider can provide guidance on this.  Continued Protection: Remember, stopping PrEP means losing a level of protection against HIV. Condom use and other prevention strategies become even more important if you decide to stop PrEP.  Future Use: If you stop PrEP but your circumstances change, you can restart it in the future. This should be done under the supervision of a healthcare provider, with an HIV test before restarting to make sure you are still HIV-negative.  Regular Testing: Regular HIV testing and screening for other sexually transmitted infections is still important, even if you decide to stop PrEP.  Remember that stopping PrEP should be a considered decision based on your individual risk, changes in your personal circumstances, and in consultation with your healthcare provider. |  |
| 1. I want to stop PrEP; How many days must pass after the last relationship at risk to be able to suspend it? | |
| If you're considering stopping PrEP (Pre-exposure Prophylaxis), it's critical to have a conversation with your healthcare provider. They can provide the best advice tailored to your specific circumstances and risk factors.  However, current guidelines from the CDC suggest that individuals should continue taking PrEP for 28 days after their last potential exposure to HIV. This is based on the longest estimated time frame for the virus to become detectable after exposure (the "window period") in a majority of people.  Keep in mind, these are just general guidelines and the specifics could vary depending on a variety of factors, including the type of exposure and the exact PrEP regimen being used.  It's also important to understand that stopping PrEP would leave you without this form of protection against HIV if you engage in behavior that puts you at risk for exposure to the virus. If you're planning on continuing activities that put you at higher risk for HIV, you may want to reconsider stopping PrEP, or explore other strategies for reducing your risk. Again, the best course of action would be to have a detailed discussion with your healthcare provider. |  |
| 1. What does it means PreP on demand? | |
| "PrEP on demand," also known as "event-driven PrEP" or "intermittent PrEP," is a way of taking HIV pre-exposure prophylaxis (PrEP) medication based on when you plan to have sex, rather than taking it every day.  The on-demand regimen, primarily designed for men who have sex with men, involves taking a double dose (two pills) of the medication 2-24 hours before anticipated sexual activity, then, if sex occurs, taking one pill 24 hours after the double dose and one more pill 24 hours later. If sexual activity lasts for several days, one pill should be taken each day until 48 hours after the last sexual activity.  This approach requires a good understanding of your sexual behavior and being able to predict when you will have sex. It also requires careful adherence to the dosing schedule around the time of sexual activity.  While the daily PrEP regimen is recommended for all people at high risk of getting HIV, regardless of gender or sex act, PrEP on demand is so far only recommended for men who have sex with men, based on the results of the clinical trials. Always consult with your healthcare provider to determine which regimen is best suited for you.  As of my knowledge cutoff in September 2021, the medications approved for on-demand use are limited, and in some places, this may only include Truvada (emtricitabine/tenofovir disoproxil fumarate). Always check with a healthcare professional or the latest resources to get the most current information. |  |
| 1. I am a woman, may I use PrEP? | |
| Yes, women can and do use pre-exposure prophylaxis (PrEP) to prevent HIV infection. PrEP is a medication that is taken daily to reduce the risk of acquiring HIV, and it is recommended for anyone who is at a high risk of getting HIV, including women who:  Have a sexual partner who is HIV-positive   - Have multiple sexual partners, particularly if condoms are not consistently used - Have a sexual partner who is at risk for getting HIV (for example, a partner who injects drugs or has multiple partners) - Have recently been treated for a sexually transmitted infection - Inject drugs and share needles or other equipment   For PrEP to be effective, it must be taken consistently and correctly as prescribed by a healthcare provider. PrEP is even more effective when it is combined with other methods to prevent HIV infection, such as condom use.  In addition to prescribing PrEP, your healthcare provider will also conduct routine check-ups, which usually include HIV tests, side effect screenings, and assessments of kidney function due to the medication's potential effect on the kidneys.  It's also important to note that while PrEP is highly effective in reducing the risk of HIV transmission through sex, it does not protect against other sexually transmitted infections (STIs). Furthermore, PrEP does not prevent pregnancy. Therefore, if you are sexually active and want to avoid pregnancy, you should still use contraception.  As of my knowledge cutoff in September 2021, the daily use of the drug Truvada (emtricitabine/tenofovir disoproxil fumarate) and Descovy (emtricitabine/tenofovir alafenamide) are approved for use as PrEP in women. Always check with a healthcare professional or the latest resources to get the most current information. |  |
| 1. I am a woman, may I use PreP on demand? | |
| As of my knowledge cutoff in September 2021, "PrEP on demand," also known as "event-driven PrEP" or "intermittent PrEP," is generally not recommended for women.  PrEP on demand involves taking a dose of PrEP medication before and after sex, rather than on a daily basis. This method has been studied and found to be effective in preventing HIV among men who have sex with men. However, studies suggest that for PrEP to be effective in vaginal sex, it must be taken consistently every day. This is because the tissues in the vagina may not achieve protective drug levels as quickly as rectal tissues.  This advice could change as more research is done. Therefore, it is important to consult with a healthcare provider for the most up-to-date information and to find out which method of using PrEP is most suitable for your personal circumstances. Always remember that PrEP should be used in combination with other protective measures, such as using condoms, to reduce the risk of other sexually transmitted infections and unwanted pregnancy. |  |
| 1. Does it exist a pill protecting both from HIV and pregnancy exist? | |
| As of my training cut-off in September 2021, there isn't a single pill that protects against both HIV and pregnancy.  For pregnancy prevention, there are various forms of contraception including pills, injections, implants, intrauterine devices (IUDs), and condoms.  For HIV prevention, a medication called pre-exposure prophylaxis (PrEP) can be used by people who are HIV-negative but at high risk of HIV infection. PrEP needs to be taken consistently to provide effective protection against the virus.  However, it's important to note that while PrEP and contraceptives can prevent HIV infection and pregnancy respectively, they do not protect against other sexually transmitted infections (STIs). Condoms are currently the only form of protection that can prevent both pregnancy and most STIs, including HIV, when used correctly.  If you are considering methods for pregnancy prevention and HIV protection, it's recommended that you have a discussion with a healthcare provider. They can provide advice tailored to your individual circumstances and needs, including the most up-to-date and accurate information.  Remember, this information is accurate up to my last training data in September 2021, and there may have been advancements or changes in the field since then. Always consult with a healthcare provider for the most current and personal advice. |  |
| 1. I am a transgender woman, may I use PreP on demand? | |
| As of my knowledge cutoff in September 2021, there has not been sufficient research to definitively recommend "PrEP on demand," also known as "event-driven PrEP" or "intermittent PrEP," for transgender women.  PrEP on demand involves taking a dose of PrEP medication before and after sex, rather than on a daily basis. This method has been primarily studied in men who have sex with men, and it has been found to be effective in this group.  For transgender women, whether engaging in vaginal or anal sex, daily PrEP is typically recommended to ensure protective drug levels. This is especially important for transgender women taking feminizing hormones, as there has been limited research on potential interactions between PrEP medications and hormone therapy.  Transgender women have been underrepresented in PrEP research, and more studies are needed to provide clear guidelines on the use of event-driven PrEP in this population.  Therefore, it's important for transgender women to consult with a healthcare provider for the most up-to-date information and to find out which method of using PrEP is most suitable for their personal circumstances. Always remember that PrEP should be used in combination with other protective measures, such as using condoms, to reduce the risk of other sexually transmitted infections. |  |
| 1. Is it more efficient to continuous PrEP or PrEP on demand? | |
| As of my knowledge cutoff in September 2021, the efficiency of PrEP, whether it's taken daily (continuous PrEP) or on demand (event-driven PrEP), largely depends on adherence to the medication schedule and the type of sexual activity.  Continuous PrEP, where the medication is taken daily, has been proven to be highly effective in preventing HIV among all populations when taken as prescribed. It allows for a constant level of the drug to be maintained in the body, providing continuous protection against the virus.  PrEP on demand, which involves taking the medication before and after sex, has also been found to be effective, but this strategy has mostly been studied in men who have sex with men. In these studies, PrEP on demand was nearly as effective as daily PrEP, provided the regimen was strictly followed.  However, PrEP on demand might not be as effective for people who have vaginal sex, because the drugs used in PrEP achieve protective levels more quickly in rectal tissue than in vaginal or cervical tissues. This means that daily dosing might be necessary for these individuals to maintain the drug levels needed for protection.  The choice between continuous PrEP and PrEP on demand largely depends on individual lifestyle, the frequency and type of sexual activity, and the ability to plan for sexual activity. It's also worth noting that neither method protects against other sexually transmitted infections, so they should be used in combination with other protective measures, such as condoms.  As always, it's recommended to consult with a healthcare provider to determine the best approach to PrEP for each individual, based on their specific circumstances and needs. Future research may also provide more insights into the comparative efficiency of these strategies. |  |
| 1. How may I know if I am eligible to assume PrEP? | |
| The decision to start PrEP (Pre-Exposure Prophylaxis) should be made in consultation with a healthcare provider who can assess your individual risk factors for acquiring HIV.  According to the U.S. Centers for Disease Control and Prevention (CDC), PrEP may be considered for people who are HIV-negative and fall into one or more of the following categories:  Individuals in a sexual relationship with a partner who has HIV, particularly if the partner has not been on consistent HIV treatment or has a detectable viral load.  Gay or bisexual men who have had anal sex without a condom or have been diagnosed with a sexually transmitted infection (STI) in the past six months.  Heterosexual men or women who do not regularly use condoms during sex with partners of unknown HIV status who are at substantial risk of HIV infection (for example, people who inject drugs or women who have bisexual male partners).  People who have injected drugs in the past six months and have shared injection equipment or have been in drug treatment in the past six months.  This list is not exhaustive, and there may be other factors or circumstances that could make you a good candidate for PrEP.  If you think you may benefit from PrEP, discuss it with your healthcare provider. They will also conduct tests to confirm you are currently HIV-negative and that your kidneys are functioning well (since the medications used in PrEP can affect kidney function), before prescribing PrEP. Regular follow-up appointments will also be needed to check for possible side effects, test for STIs, and test for HIV.  Remember, PrEP is only one part of a comprehensive HIV prevention strategy. It should be used in combination with other methods, such as condom use and safer injecting practices, to reduce the risk of acquiring HIV. |  |
| 1. Can I take recreational drugs when on PrEP? | |
| While it's not recommended to take recreational drugs due to the many health risks associated with their use, it's important to note how they might interact with pre-exposure prophylaxis (PrEP) if they are being used.  PrEP medication itself does not have known interactions with most recreational drugs. However, this does not mean it's safe to use recreational drugs while on PrEP. The behaviors sometimes associated with recreational drug use can present risks. For instance, drug use can:  Interfere with Medication Adherence: If you're using recreational drugs, they might affect your ability to take PrEP consistently, which is essential for its effectiveness in preventing HIV.  Increase Risky Behavior: Some drugs can lower inhibitions, leading to riskier sexual behavior, which could increase your chances of contracting HIV or other sexually transmitted infections (STIs).  Cause Health Complications: Recreational drugs can have harmful effects on your health, particularly your mental health, liver, heart, and brain. Some of these effects might be exacerbated if you're also taking PrEP.  Remember, it's essential to discuss recreational drug use with your healthcare provider to understand potential interactions and risks. This conversation is important for your safety, and your provider is there to help you without judgment. As of my training cut-off in September 2021, it's important to have these conversations and to remember that recreational drug use carries significant risks.  Please note that newer information might be available if you're reading this after my training cut-off. Always consult with a healthcare provider for the most current and personal advice. |  |
| 1. I use injective drugs, can I still take PrEP? | |
| Yes, people who inject drugs can and should still consider taking pre-exposure prophylaxis (PrEP) if they are at high risk for HIV. In fact, the use of PrEP is recommended by the World Health Organization and the Centers for Disease Control and Prevention for people who inject drugs and are at high risk for HIV infection.  People who inject drugs can be at high risk of HIV infection through the sharing of needles, syringes, or other drug injection equipment. PrEP can provide a high level of protection against HIV when taken consistently and correctly.  It's essential that people who use injectable drugs also have access to comprehensive harm reduction services, including sterile injection equipment, opioid substitution therapy, and testing and treatment for hepatitis and other sexually transmitted infections.  As always, the decision to start PrEP should be made in consultation with a healthcare provider, who can provide advice tailored to the individual's specific situation and needs. Regular follow-up, including repeat HIV testing, monitoring for side effects, and adherence counseling, is a critical part of PrEP use.  Please remember that my training includes information available up until September 2021, and there may have been advancements or changes in the understanding of this subject since that time. Always consult with a healthcare provider for the most current and personal advice. |  |
| 1. What should I do if I just have had an at-risk sexual behavior? | |
| If you believe you have recently (within the last 72 hours) been exposed to HIV during sex, through sharing needles, or if you've been sexually assaulted, it's important to reach out to a healthcare provider immediately. They may suggest a course of medication known as Post-Exposure Prophylaxis (PEP).  PEP is a month-long treatment that can prevent HIV infection after the virus has entered a person's body, and it should be started as soon as possible after the exposure - ideally within 72 hours.  After potential exposure to HIV:  Seek Medical Attention Immediately: Reach out to your doctor, an emergency room, or an urgent care clinic to discuss PEP.  Start PEP: If your healthcare provider determines that you're a candidate for PEP, you should start the regimen as soon as possible and will need to take it for 28 days.  Get Tested for HIV: You'll need to get tested for HIV at the time of the potential exposure, and again at four to six weeks, three months, and six months after the potential exposure.  Follow Up: Follow up with your healthcare provider throughout and after the treatment process to monitor any side effects or complications, and to ensure the treatment is working.  Prevent Future Exposures: Discuss with your healthcare provider about preventive measures like Pre-Exposure Prophylaxis (PrEP), and consistently use condoms to lower your risk of HIV infection.  Remember, PEP is not 100% effective. It's not intended for regular or frequent use and it does not replace regular use of other HIV prevention methods like condoms or PrEP.  Consult your healthcare provider for any questions or concerns you have about your risk or HIV prevention strategies. |  |
| 1. What is postexposure prophylaxis (PEP)? | |
| Post-Exposure Prophylaxis (PEP) is a course of HIV medications which is taken after potential exposure to HIV to prevent becoming infected. It's used in emergency situations and must be started within 72 hours after a recent possible exposure to HIV. If you think you’ve recently been exposed to HIV during sex, through sharing needles, or if you’ve been sexually assaulted, you should contact your health care provider immediately or go to an emergency room to discuss the option of PEP.  PEP involves taking antiretroviral drugs for 28 days. It's important to know that PEP should be used only in emergency situations and is not meant for regular use by people who may be exposed to HIV frequently.  For those who are at ongoing risk for HIV, Pre-Exposure Prophylaxis (PrEP), a daily medication, is usually recommended. It's always important to use other protection methods like condoms during sex and not sharing needles, even when on PEP or PrEP. |  |
| 1. What it the efficacy of PEP? | |
| Post-Exposure Prophylaxis (PEP) is highly effective in preventing HIV infection if it's started as soon as possible, ideally within 72 hours of exposure, and taken consistently for the full 28-day course.  However, it's not 100% effective. The exact efficacy can depend on several factors, including the type of exposure, the time between exposure and starting PEP, and how consistently the medication is taken.  Keep in mind that PEP is a form of emergency intervention, not a replacement for ongoing preventive measures like regular use of condoms, safe injection practices, or Pre-Exposure Prophylaxis (PrEP) for people with regular high-risk exposures.  If you believe you've been exposed to HIV, it's important to seek medical attention immediately to discuss the possibility of starting PEP. The sooner it's started after exposure, the more likely it is to be effective. |  |
| 1. I got stung myself with a used syringe on the beach; what should I do? | |
| If you accidentally prick yourself with a used needle or syringe, it's important to take immediate action due to the risk of potential infection, including HIV:   - Wash the area: Rinse the site immediately with warm water and soap to clean the wound and help reduce the risk of infection. - Don't squeeze: Try not to squeeze or forcefully bleed the wound as this can potentially push pathogens further into your bloodstream. - Seek medical attention: As soon as you can, visit a healthcare provider or an emergency room. Inform them about the incident in detail. This includes where you were, what you were doing, and any other specifics about the needle or syringe if available. - Post-Exposure Prophylaxis (PEP): Depending on the risk assessment, your healthcare provider may recommend starting PEP, which involves taking antiretroviral drugs for 28 days to prevent HIV infection. - Follow-up testing: You will likely need to undergo blood tests immediately and at intervals afterwards to check for infections such as HIV, hepatitis B, and hepatitis C. - Vaccination: If you are not already vaccinated, a Hepatitis B vaccination may be recommended.   Remember, it's crucial to seek medical attention immediately after the incident, as PEP is most effective when started within 72 hours. |  |
| 1. I got stung with a needle after having used on a person with unknown serostatus; what should I do? | |
| If you've had a needlestick injury after using a needle on a person of unknown serostatus (you don't know if they have HIV, Hepatitis B, or Hepatitis C), follow these steps:   - Immediate care: Wash the area with soap and warm water immediately. Don't squeeze the wound or try to force it to bleed. - Medical attention: Seek medical attention immediately. This is a medical emergency. Go to an emergency room if you can't see your regular healthcare provider right away. - Assessment: Your healthcare provider will assess your risk of infection based on details of the exposure and the source patient's infection status, if known. - Post-Exposure Prophylaxis (PEP): If the risk of HIV infection is deemed significant, your healthcare provider may recommend a course of antiretroviral drugs (PEP) to prevent HIV infection. This needs to be started within 72 hours after the exposure and ideally as soon as possible. - Follow-up: Over the following weeks to months, you'll need to have blood tests to check if you've been infected with HIV, hepatitis B, or hepatitis C. Follow your healthcare provider's instructions on when and how often to get tested.   Remember, prompt action is key when dealing with potential exposures to HIV and other bloodborne pathogens. The sooner you seek medical attention, the more options you'll have to potentially prevent infection. |  |
| 1. I got stung with a needle after having used on a person with HIV; what should I do? | |
| If you've been accidentally pricked with a needle that has been used on a person known to be HIV positive, here's what you should do:   - Immediate care: Clean the area immediately. Wash the wound with soap and warm water. Do not squeeze the wound or try to force it to bleed. - Medical attention: Seek medical attention immediately. This is considered a medical emergency. Go to the nearest emergency department as soon as possible. - Post-Exposure Prophylaxis (PEP): The healthcare provider will likely recommend starting PEP as soon as possible, ideally within 1-2 hours and no later than 72 hours after the exposure. PEP involves taking a combination of HIV medications for 28 days to prevent HIV infection. - Follow-up: You will need to have follow-up blood tests to check for HIV infection. These tests are typically done immediately after the exposure, and then again at 6 weeks, 12 weeks, and 24 weeks after the exposure. - Mental health support: Experiencing a needlestick injury can be distressing. You may feel anxiety or fear about the risk of HIV infection. Consider seeking support from a mental health professional or a support group for healthcare workers who've experienced occupational exposures.   Remember, the key is to act immediately. The sooner PEP is started, the more likely it is to be effective. PEP is not 100% effective, but it significantly reduces the risk of HIV transmission after an exposure. |  |
| 1. Should a person who has been sexually assaulted start PEP? | |
| Yes, Post-Exposure Prophylaxis (PEP) should be considered in cases of sexual assault due to the potential risk of exposure to HIV. The decision to start PEP should be made as quickly as possible, ideally within 72 hours of the potential exposure. Here are some important steps that a person who has been sexually assaulted should take:   - Immediate care: Seek medical attention immediately. This is a medical emergency. - Medical attention and assessment: A healthcare provider will assess the individual's risk for HIV and other sexually transmitted infections (STIs) based on the specifics of the assault. They will also consider the local epidemiology of HIV and STIs. - Post-Exposure Prophylaxis (PEP): If the healthcare provider determines that there is a substantial risk for HIV transmission, they will likely recommend starting PEP as soon as possible, ideally within 72 hours after the exposure. PEP involves taking a combination of HIV medications for 28 days to prevent HIV infection. - Follow-up care: Individuals who start PEP will need to have follow-up blood tests to check for HIV infection. These tests are typically done immediately after the exposure, and then again at 6 weeks, 12 weeks, and 24 weeks after the exposure. - Support services: Being sexually assaulted is a traumatic experience. Accessing support from mental health professionals, support groups, and other services designed for survivors of sexual assault can be crucial for recovery.   It's important to remember that PEP should be part of a comprehensive post-assault care that includes treatment for other potential STIs, pregnancy prevention, and appropriate psychological and social support. Also, a healthcare provider might recommend Pre-Exposure Prophylaxis (PrEP) as an ongoing prevention strategy after completing PEP, depending on the person's circumstances. |  |
